# The use of smart surveillance technologies for suicide prevention in public spaces: a professional stakeholder survey from the United Kingdom

**DOI:** 10.1186/s12889-026-26739-0

**Published:** 2026-03-19

**Authors:** Laura Joyner, Bethany Cliffe, Jay-Marie Mackenzie, Elizabeth Pettersen, Ian Marsh, Penny Phillips, Lisa Marzano

**Affiliations:** 1 Department of Psychology, Middlesex University, London, UK; 2 Department of Psychology, University of Westminster, London, UK; 3 Samaritans, Ewell, UK; 4 Faculty of Medicine, Health and Social Care, Canterbury Christ Church University, Canterbury, UK; 5 NSPA Lived Experience Advisory Group, Ewell, UK

**Keywords:** Suicide, Suicide prevention, Hotspots, Surveillance technology, Smart surveillance, CCTV, Artificial intelligence, Real-world application, Human factors, Public safety

## Abstract

**Background:**

Around a third of suicides in the United Kingdom occur in public spaces, such as on the railways, at bridges, or coastal locations. Increasingly, the use of Artificial Intelligence and other smart technologies are being proposed as a means of optimising or automating aspects of the surveillance process in these environments. Yet relatively little is known about how they are being used for suicide prevention and the realities of deploying these systems in public spaces.

**Methods:**

108 professional stakeholders across the UK completed an online survey to understand how smart surveillance technologies are being deployed across different types of public spaces to (also) prevent suicides. Through a series of multiple choice and open-ended questions, participants were also asked about the perceived benefits, limitations, and biggest barriers of implementing these technologies for suicide prevention reasons.

**Results:**

72 examples of smart surveillance technology were identified, with around two-thirds at “high-risk” locations. Motion-activated CCTV, cameras with AI analytics, and Automatic Number Plate Recognition (ANPR) cameras were the most commonly identified technologies. More than half of systems alerted a control room when activated (58%), and the majority of all identified systems (80%) initiated a human-led response. Qualitative analysis suggests that these technologies can help guide real-time or future rescue responses. However, the importance of ensuring technology met the needs of a location was raised, with many originally designed for other purposes (e.g., crime prevention). Furthermore, several participants indicated technology alone could not prevent suicides, and felt a human response was still required. This, however, presented challenges such as the feasibility of delivering rapid responses. Barriers to installation and other challenges, including ethical and legal concerns, were also raised.

**Conclusions:**

The present findings suggest that smart surveillance technologies have the potential to aide suicide prevention efforts but are unlikely to replace other measures. The findings highlight the importance of engaging with other stakeholders, including staff who lead the response or work with the systems day-to-day and people with lived experience of suicide. Furthermore, environmental factors, existing infrastructure and the processes surrounding the use of these tools may also influence their effectiveness as a suicide prevention measure when deployed in real-world settings.

**Supplementary Information:**

The online version contains supplementary material available at 10.1186/s12889-026-26739-0.

Efforts to prevent suicide in public spaces have emerged as important elements within international suicide prevention strategies [1, 2]. In 2022, approximately 6,500 suicides were recorded across the United Kingdom [3–5]. Previous estimates suggest around a third of these deaths occur in public places, including bridges, cliffs, and the railways [6]. Not only do these deaths have devastating consequences for families and friends; witnesses, including those working at the location, may also be impacted [7–9]. Furthermore, there can be substantial financial implications for organisations responsible for a location. For instance, the annual cost of suicides to the British rail industry is estimated to be around £67 million [10]. As such, both public and private organisations are often motivated to work collectively with an aim of preventing suicide in public spaces.

A range of strategies have been recommended to help prevent suicide in public locations, including restricting access to means, increasing opportunity for human intervention, encouraging help-seeking, and responsible media reporting [11]. While some interventions involve physical or educational measures, it is thought surveillance could play a useful role in suicide prevention by aiding responses. However, the evidence around the effectiveness of surveillance technologies for preventing suicides remains mixed [12]. Some research suggests the presence of CCTV cameras (often alongside other measures) may be helpful at preventing some suicides at high-risk locations, such as on the railways [13] or at bridges [14, 15]. Yet, other studies have found no evidence of CCTV cameras having any such preventative effect [16–18]. The reasons for this are currently unclear. However, traditional surveillance systems notably require high levels of attention for extended periods of time from human operators, who sometimes must also monitor live footage captured across multiple environments [19]. These practical challenges could potentially impact ability to detect and respond to events in real-time.

However, in recent years there has been growing interest in the use of “smart” surveillance systems to support suicide prevention efforts. These technologies are designed to automate aspects of the surveillance processes (e.g., through the use of Artificial Intelligence), and may therefore help alleviate some of the monitoring burden from human operators. Yet, little is known about whether systems are actually being used for suicide prevention in the real-world, or the suitability of these tools to support suicide prevention efforts in public spaces (in terms of both practicalities and any ethical challenges): this is something we seek to address with this paper.

## Background

### Smart surveillance technologies

Smart surveillance technologies (SSTs) enhance or fully-automate surveillance processes through the extraction and interpretation of information received from devices and / or sensors (see Langheinrich et al., [20] for a detailed overview). Increasingly, Artificial Intelligence (AI) is also integrated into surveillance systems as a means of analysing live data; such as computer vision processes used to detect and classify objects, or identify patterns in video footage (a glossary of key terms used throughout this paper can be found in Table 1). The way in which SSTs detect and respond to “events” can therefore vary greatly, and their ability to do this is in part informed by how they are designed. For example, passive infrared sensors and analytics applied to a CCTV feed are both approaches which could be used to detect the presence of a person somewhere where they shouldn’t be. However, decisions about which method would be most appropriate to use may be informed by environmental and situational characteristics, for example, whether monitoring needs to happen in the dark.

**Table 1:** Glossary of key terms

Term	Definition
Smart Surveillance	Smart surveillance systems harness data from one or more sensors (e.g., Visible Camera), and apply tools and functions (e.g., Artificial Intelligence) to collect, process and use the captured information in ways which support the partial or full automation of surveillance processes ^a^. This may include systems which help detect individuals entering into zones of interest (e.g., “Virtual Fences”, “(Perimeter) Intrusion Detection Systems”) or their presence within a high-risk area (e.g., “Proximity Warning System”, “Obstacle Detection”).
Use case	A specific goal that an SST could (or is used to) support (e.g., to prevent trespass).
Computer Vision	An area of computer science, where artificial intelligence is used to extract information from visual images (e.g., videos).
Video Analytics	Assists the surveillance process by automatically processing video analysis on footage using combinations of computer vision techniques ^b^
Virtual Fences	A virtual “barrier” drawn within an environment that is invisible to the human eye ^c^. May be produced using sensors within the environment itself (e.g., Active Infrared, Bistatic Microwave) or via live camera footage using AI-based Video Analytics (e.g., Trajectory Analysis). When breached, activates automated processes to alert human operators and/or generate a technology-based response (e.g., activates warning message).
Examples of Sensors	
Active Infrared	Have the capability to both transmit and receive infrared. One use is as a “Virtual Fence”, where devices producing an invisible barrier of infrared beams are installed in an environment. When beams are disrupted (e.g., due to a person walking through) the system would then trigger the programmed response ^c^.
Passive Infrared (PIR)	Detect (but do not transmit) infrared light emitted from warm objects. Systems using PIR sensors therefore identify energy changes within an environment ^c^ (e.g., human presence). May be used to activate a system (e.g., alarms, CCTV, lighting) or automate the direction of robotic Pan-Tilt-Zoom cameras (i.e. towards energy source).
Microphonic Sensor	These may be attached to barriers (e.g., fences) or buried underground to detect vibrations at a frequency associated with a person entering the environment ^c^.
Radar	Radio Detection and Ranging (RADAR) transmits and receives radio wave pulses to determine distances. Can be used to detect individuals within large environments and / or guide the direction of a Pan-Tilt-Zoom camera to automate tracking.
LiDAR	Light-Detection and Ranging (LiDAR) is an active remote sensing technology, where scanning lasers emit pulses of light and receive the reflecting “echo” to measure distance. Greater accuracy and image resolution than radar, but shorter range. Can be used in different ways, for instance to help detect physical changes in a pre-determined zone (e.g., at level crossings) or to produce 3D images of a space (which in turn, can be analysed using AI).
BLE Beacon	Bluetooth Low Energy (BLE) Beacons are wireless, low-power devices which broadcast signals detectable by BLE-enabled devices such as smartphones. Static placement of multiple beacons can be used to produce positioning systems, identifying locations of BLE-enabled devices in an area ^d^.
Examples of AI Tools & Techniques	
Automatic Number Plate Recognition (ANPR)	ANPR (also known as License Plate Recognition; LPR) produces machine-readable vehicle number-plate data (e.g., text string) from an image or video. Usually involves a process incorporating a series of techniques for (1) number-plate extraction, (2) character segmentation, and (3) character recognition ^e^. In the United Kingdom (UK), approved organisations (e.g., Police) may connect ANPR to a restricted national database allowing the system to flag vehicles of interest, including those of vulnerable missing persons (45).
Moving Object Detection	Detects movement within defined area in image, for instance, by identifying changes in pixels across video frames.
Trajectory Analysis	Analyses paths of people or moving objects across time to detect abnormal events, including pacing ^f^ and lingering ^g^. Where regional information about the environment is defined, TA can also be used to detect entry into high-risk zones ^b^.
Pose Estimation	Detects the location of key joints on a human body (e.g., “right shoulder”) and their configuration in relation to one another to analyse pose. This can help a system identify, for instance, a person crouching in an area where it is usual to stand ^h^ or a hanging attempt ^i^. Can be applied for human activity recognition (see below), gait analysis and fall detection, amongst others.
Human Action Recognition (HAR)	HAR algorithms aim to automatically detect activities predefined by the system user ^b^. These activities are classified into three types of categories; (1) gestures (motions, e.g., raising arm), (2) actions (series of gestures, e.g., running) and (3) interactions (between humans and/or objects; e.g., fighting, abandoning bag).
Video Anomaly Detection (VAD)	Aims to detect abnormal behaviour in video footage. Some models trained using existing footage pre-categorised into “normal” and / or “abnormal” events. Alternatively, an unsupervised model may learn to detect “novel events” from unlabelled footage based their relative probability of occurrence overall ^b^.
Live Facial Recognition (LFR)	Compares an existing database of faces (e.g., containing persons of interest) against live camera feeds and alerts operators when matches are found. This process usually involves three steps: (1) face detection (i.e. finding human faces within an image), (2) feature extraction (i.e. information about prominent facial features), and (3) face recognition (i.e. against existing database) ^j^.
Examples of System Responses	
Audible deterrents	Recorded warning messages and / or alarms automatically emitted by the system when activated.
Live audio system	Speakers allowing human operators to issue audible messages (1-way) or instigate conversation with individuals at site (2-way).
Visual alerts	Lights (e.g., LEDs, strobe) activated by the system during event.
Video timestamps	A digital marker indicating time of key events in video footage, which may be automatically generated by system.
Online Interceptive Tool	These tools present digital interventions to internet users searching for harmful content while using a device or specific WiFi network where the tool is already deployed.

^a^Langheinrich M, Finn R, Coroama V, Wright D. Quo vadis Smart Surveillance? How smart technologies combine and challenge democratic oversight. In: Gutwirth S, Leenes R, De Hert P, editors. Reloading data protection: multidisciplinary insights and contemporary challenges. Dordrecht: Springer Netherlands; 2014. p. 151–82

^b^Zhang T, Aftab W, Mihaylova L, Langran-Wheeler C, Rigby S, Fletcher D, et al. Recent advances in video analytics for rail network surveillance for security, trespass and suicide prevention—a survey. Sensors. 2022 Jan;22(12):4324

^c^National Protective Security Authority. Perimeter Intrusion Detection [Internet]. UK Government; [Updated 2023 Dec 13; cited 2024 Aug 19]. Available from: https://www.npsa.gov.uk/perimeter-intrusion-detection-pids-0

^d^Spachos P, Plataniotis K. BLE beacons in the smart city: Applications, challenges, and research opportunities. IEEE Internet of Things Magazine. 2020 Mar;3(1):14-8

^e^Lubna, Mufti, N., & Shah, S. A. A. (2021). Automatic number plate recognition: a detailed survey of relevant algorithms. Sensors, 21(9), 3028

^f^Onie, S., Li, X., Liang, M., Sowmya, A. and Larsen, M.E., 2021. The use of closed-circuit television and video in suicide prevention: narrative review and future directions. JMIR mental health, 8(5), p.e27663

^g^Li X, de Belen RA, Sowmya A, Onie S, Larsen M. Region-based trajectory analysis for abnormal behaviour detection: a trial study for suicide detection and prevention. In International Conference on Pattern Recognition 2022 Aug 21 (pp. 178-192). Cham: Springer Nature Switzerland

^h^Li X, Onie S, Liang M, Larsen M, Sowmya A. Towards building a visual behaviour analysis pipeline for suicide detection and prevention. Sensors. 2022 Jan;22(12):4488

^i^Bouachir W, Gouiaa R, Li B, Noumeir R. Intelligent video surveillance for real-time detection of suicide attempts. Pattern Recognition Letters. 2018 Jul 15;110:1-7

^j^Kortli Y, Jridi M, Al Falou A, Atri M. Face recognition systems: a survey. Sensors. 2020 Jan 7;20(2):342.

When an SST then detects an “event” its response may also vary. For example, the system may send alerts to pre-defined individuals (e.g., staff) who may then choose to mobilise a human-led response. Alternatively, SSTs may operate as standalone interventions, for instance, by producing a pre-recorded audible deterrent at the location itself. Arguably, these outcomes have important implications for understanding how, when and why these systems may be effective at preventing suicides, including how people (e.g., staff, members of the public) may interact with them.

### Uses for smart surveillance technologies in suicide prevention

In recent years, industry reports have identified SSTs as a potential tool to aid suicide prevention efforts on the railways [21, 22], at multi-storey carparks [23] and at bridges [24]. Guidance on preventing suicides in public spaces by Public Health England also identify SSTs as being a potentially useful measure to increase opportunity for human intervention at high-risk locations [11]. However, it is also stressed that surveillance technology alone is not a solution. While SSTs are often viewed as a way to support suicide prevention efforts by monitoring high-risk areas (e.g., bridge barriers, railway tracks), there is evidence of them also being proposed or used to support surveillance across larger environments, help locate or identify specific individuals, and identify certain behaviours that may indicate risk. The following sections briefly outline how SSTs may support these different approaches.

#### Monitoring high-risk areas

One approach is to use SSTs to monitor for people entering into high-risk areas (e.g., railway tracks) primarily to nitiate the deployment of a rescue response. a rescue response. For example, infrared beams have previously been used in suicide prevention efforts to detect people entering the tunnels at metro stations [25] or passing over bridge barriers [26]. Other sensors (e.g., tension-wire sensors) have been used to detect people climbing barriers at coastal locations [27] and at bridges [28]. Alternatively, video analytics may be applied to real-time camera footage to detect movement (e.g., by detecting changes across video frames) or identify that a ‘person’ is ‘entering’ a pre-defined area (e.g., using trajectory analysis and regional information), and can even time how long they remain there (i.e., ‘linger’). Similar approaches have been used in systems piloted in the real world for 'safeguarding' purposes at metro stations, with the systems designed to alert staff to unauthorised persons entering or remaining in defined areas [29, 30]. There are also SSTs which operate as “standalone interventions” and, instead of notifying a person, may produce a technological response (e.g., an alarm or visual alert) at the location. For example, SSTs featuring motion sensitive lighting have been deployed at a bridge in Korea [31] and at a rail station in Denmark [32] as part of suicide prevention efforts. Moreover, while the use of sensor-activated sound warnings have been tested on the railway as means of deterring trespass [33, 34], it is unclear whether such an approach has been used in the real-world for suicide prevention [12].

There is, however, limited evidence to suggest SSTs designed to monitor high-risk environments are effective at preventing suicides [12]. This is despite some studies observing an increase in the number of rescue attempts or police call outs following installation of surveillance technologies [18, 26, 35]. This highlights the importance of understanding the real-world practicalities that may then impede suicide prevention efforts on the ground. Additionally, false alarms can also present an issue with these types of SSTs, which others have suggested could potentially be reduced by combining sensors with AI (e.g., using thermal cameras to detect the presence of a human and video analytics to then filter out irrelevant objects such as trains and video analytics to then filter out irrelevant objects such as trains [36]). Understanding key learnings around what has and has not worked from previous real-world deployments of these devices may arguably be useful for those looking to deploy them in suicide prevention efforts in the future.

#### Surveillance of large environments

Certain environments may present challenges for surveillance due to their scale, and therefore alternative types of SSTs may be required to support suicide prevention efforts. For example, autonomous and piloted Unmanned Arial Vehicles (UAVs, e.g., Drones) have been reported as being used in suicide prevention efforts at one forest outside of the UK [37]. The possibility of drones being used to support suicide prevention efforts has also been identified by rail industry professionals internationally [22, 38, 39]. There are, however, strict regulations in the UK around where commercial drones can be flown and who is qualified to pilot them [40] Which could also present barriers for their use in suicide prevention.

Location determination technologies designed to identify and track the location of individuals via their personal devices (e.g., mobile phones) are another approach for monitoring large-scale environments. However, their use can also attract privacy concerns [41]. Whether they have been used in suicide prevention efforts in the real world is unclear, but such technologies have previously been explored as a surveillance tool in safety research. For example, systems utilising Bluetooth Low Energy (BLE) Beacons have been tested as a way of identifying when individuals are approach a potential hazard e.g., a busy street [42, 43] or lingering for too long in a dangerous area e.g., a bathroom [44]. However, as the effectiveness of these systems are dependent on individuals having a compatible device on their person (e.g., a mobile phone), their usefulness for suicide prevention in public spaces is currently unclear.

#### Identifying known persons

Some AI-driven systems may also have the potential to help locate specific individuals who have previously been flagged as being vulnerable. For example, ANPR (Automated Number Plate Recognition; also known as License Plate Recognition) can identify vehicles whose numberplates are recorded in connected databases. In the UK, ANPR is one tool that can be used by police during searches for vulnerable missing persons [45], including people for whom there are concerns around self-harm or suicide [46]. However, despite several decades of use on UK roads, little is known about the real-world practicalities for using ANPR in suicide prevention efforts. In recent years, several UK police forces (as well as some private organisations) have also begun to deploy Live Facial Recognition (LFR) to identify people on watchlists [47]. While LFR watchlists used by police can include “those who pose a risk of harm to themselves or others” [48], it is unclear whether it has been used in real-world suicide prevention efforts, or considered for this purpose.

A key factor that may influence the deployment of these types of SSTs in public spaces is public attitudes. previous work suggests the public may be more accepting of facial recognition when used in tasks such as missing persons searches [49], but may be more critical if they feel their individual privacy rights could be affected [50]. Both the use of ANPR and LFR have notably attracted concern from privacy campaigners and the public [47, 51, 52]. Concerns have also been raised about the potential risks associated with adding individuals with mental health issues to watchlists unnecessarily, including potential exclusion from the public space on this basis [47]. Despite this, relativley little is known about how professional stakeholders make sense of these kinds of ethical issues when considering whether to deploy these technologies for suicide prevention in public spaces.

#### Identifying and predicting behaviours

Previous work has identified potential patterns of behaviour that may precede suicide attempts within public settings [53, 54]. It has therefore been suggested that SSTs could use Artificial Intelligence to help identify patterns of behaviours that may be unusual within an environment or suggest a person may be in crisis (e.g., in video footage). For example, studies have looked at whether systems employing techniques such as Trajectory Analysis, Pose Estimation and Human Action Recognition may be useful for detecting unusual movements (e.g., pacing) [55] or people standing or leaning for extended periods [56] at one coastal location. Alternatively, researchers have proposed SSTs that also incorporate other event-related information when forecasting a detected individual’s “risk”, including their personal characteristics (e.g., age, gender), biometric signals (e.g., emotion recognition), and / or situational factors (e.g., how many other people are within the environment) [57]. However, while the ability to identify vulnerable persons prior to them reaching dangerous areas may appear promising, very little is known about how well such systems actually work in practice and whether they have been deployed in the real world.

### Understanding the realities of implementing smart surveillance for suicide prevention

While attention towards smart technologies has grown rapidly in recent years, the proposed use of SSTs in suicide prevention is itself not new, going back to at least the 1990s [25]. It is then somewhat notable that, over thirty years later, relatively little is known about how widely used these technologies are for suicide prevention or their actual effectiveness at preventing suicides. A lack of technological advancement is unlikely to be the reason for this, as SSTs are relatively common in day-to-day life (e.g., smart doorbells, ANPR). It may be that identifying a behaviour as complex and high-risk as a suicide attempt in real-time is itself a challenge. However, even if SSTs show promise in trials when tested against archive footage, other real-world factors may impact their use as a suicide prevention measure in real-world public locations. For example, in these complex, dynamic environments SSTs may encounter issues related to the weather [26, 36] or connectivity [58], impeding their ability to operate in real-time. Furthermore, while research suggests SSTs could help staff with locating vulnerable individuals at some locations [27], the actual availability of staff to then physically respond to incidents can itself be a challenge [59]. Therefore, while SSTs may help to automate aspects of surveillance, their compatibility with and integration alongside other processes is another important consideration.

In the present study we therefore seek to understand the ways in which SSTs are currently being used (both for suicide prevention and other purposes) in public spaces within the United Kingdom (UK), while also considering how, why, and when they may be most effective for use in suicide prevention. By surveying professional stakeholders working in suicide prevention across different types of public locations, we also aim to collate key insights on the implementation, effectiveness, and sustainability of SSTs for cross-location learning.

## Method

### Design

For this mixed-methods study, a cross-sectional survey was hosted online (via SurveyMonkey) between July 2023 – January 2024. This was for the convenience of participants (professional stakeholders located around the United Kingdom) and to provide anonymity. The survey contained both multiple-choice and open-ended questions about the use of SSTs across any locations familiar to the participant through their work (see Additional file 1).

### Participants

The survey was distributed by Samaritans to professional stakeholders in the United Kingdom (UK) whose work in some way relates to suicide prevention in public spaces. This included emailing every public health lead in the UK, as well as utilising existing networks such as the Safer Public Spaces Network (a practitioner network for people working on suicide prevention in public / high-risk locations, made up of 170 stakeholders). Samaritans also used key sector channels such as the National Suicide Prevention Alliance (NSPA) newsletter (a network of over 2,000 members) and existing contacts which included those working in areas such as emergency services, public health, community safety, and specific industries such as the railways. Several key professional bodies representing specific types of public spaces were also contacted, as well as technology and security companies known to work with UK public spaces. As part of the communication, stakeholders were encouraged to cascade the survey to colleagues and contacts who could offer further insights on this topic in order to reach as many stakeholders as possible.

To ensure the sample was as representative as possible, the survey was closed only after all regions of the UK were represented and a range of different types of locations reflected. Initially, 130 participants were recruited. However, 21 did not progress beyond demographic questions and therefore were removed from the analysis. Another participant was also removed, as their responses related to the use of technology only within secure settings. This left a total of 108 participants.

### Materials & procedure

After viewing an information page about the survey and providing consent, participants were asked to indicate which regions of the United Kingdom (UK) they worked with in their role and the Local Authority their workplace was based in. Participants were then asked a series of questions about the use and/or proposed use of smart surveillance technology (for any purpose) across three project stages: “Implemented Technology” (current and/or previously implemented), “Current Plans”, and “Discontinued Plans”. Those who indicated they were aware of projects at either stage were presented with blocks of questions about the specific site (up to two per project stage), and about the technology used (up to three per site).

For a specific site, participants were asked to indicate how the technology was being used (i.e., to prevent suicide / suicide attempts, accidental injury or death, trespass, crime / anti-social behaviour, and/or “other”) and to indicate the type of location / structure (e.g., bridge, railways, etc.). They were also asked to indicate the kind of area the site was in (i.e., urban, rural, other), level of access (i.e., public, private, other), and whether the location is known locally as a “high-risk” or “high frequency” location for suicide.

Next, participants were asked about one type of technology at said site. Firstly, they were asked to indicate the type of technology which was implemented / proposed (i.e., Automated Number Plate Recognition (ANPR), Bluetooth Low Energy (BLE) Beacons, CCTV activated by movement / proximity (MA CCTV), AI cameras, Drones, Virtual fencing / proximity warning systems, Other). They were then asked about the type of response initiated by the technology (i.e., human, standalone, unknown, and / or other) and what the technology was connected to (e.g., two-way audio systems, audible warnings / alarms, control room alert, etc.). For implemented technology, participants were also asked if the technology was visible, if it was advertised to site visitors, and whether the technology is still in use.

Participants were then asked to rate (0-100) how effective they perceived the technology to be at preventing suicides at the location. Through a series of open-text questions, they were also asked about the main benefits, risks/ limitations, and the biggest challenges/ barriers of using the technology to prevent suicides there. This was followed by further open-text questions about evaluations, costs (installation and ongoing), and financing of the technology. Participants were then asked if they were aware of any other technology at the site and if they were aware of any other locations. Where participants indicated “yes” the relevant question block was repeated. Finally, all participants were presented with a free-text box for additional comments and relevant demographic information (e.g., job role). Participants were also provided with signposting for additional support at both the start and end of the survey.

### Ethics

The study was approved on the 6th June, 2023 by the Psychology Research Ethics Committee at Middlesex University (Application 25848).

### Analysis

#### Quantitative analysis

Frequency data are presented with percentages reported to the nearest whole number. As perceived effectiveness data are non-parametric, median and IQR (where possible) are presented, and Mann-Whitney U tests used to compare differences. Graphs were produced using the ggplot2 R package.

#### Qualitative analysis

Due to the brevity of responses, open responses to questions were explored using qualitative content analysis to identify common issues reported in the data [60]. A reflexive approach was taken in that researcher subjectivity was seen as a resource rather than a bias to be neutralised. Collaboration with colleagues, transparency of analysis and triangulating with the quantitative data were used to ensure credibility and trustworthiness in the analysis.

Responses were analysed within each question, i.e., the benefits and risks/limitations were analysed separately. Responses to the question ’what is or has been the biggest challenge or barrier to using this technology to prevent suicides?’ were incorporated within the risks/limitations but were highlighted so they could still be identified. Additional comments were incorporated within the benefits or risks/limitations depending on whether the response was positive or negative. BC and IM first read all the qualitative data and BC then coded around 10% of the data inductively, developing a coding frame and discussing with IM. BC then coded the remaining data, making iterative adjustments to the coding frame where necessary throughout analysis. The codes and themes were discussed with the wider team and checked against the data to ensure it was accurately represented. They were also compared to the quantitative data to check for any insights which may have been missed. Frequency counts of each code were noted, indicating how many times a code was mentioned relative to the different types of technology and the location. The coding frame, responses and frequency counts can be found in Additional file 2.

#### Data cleaning

A small number of responses required editing during the data cleaning process. Firstly, two sets of responses relating to discontinued plans were removed as plans referred to other types of monitoring (i.e. social media monitoring and real-time surveillance). One participant provided information about implemented technology under “planned technology”, therefore responses were appropriately re-categorised. Throughout the survey, several “other” responses were recategorized to correctly record participant’s free-text responses. Details of all cleaning steps can be found in Additional file 3.

## Results

**Fig. 1:**
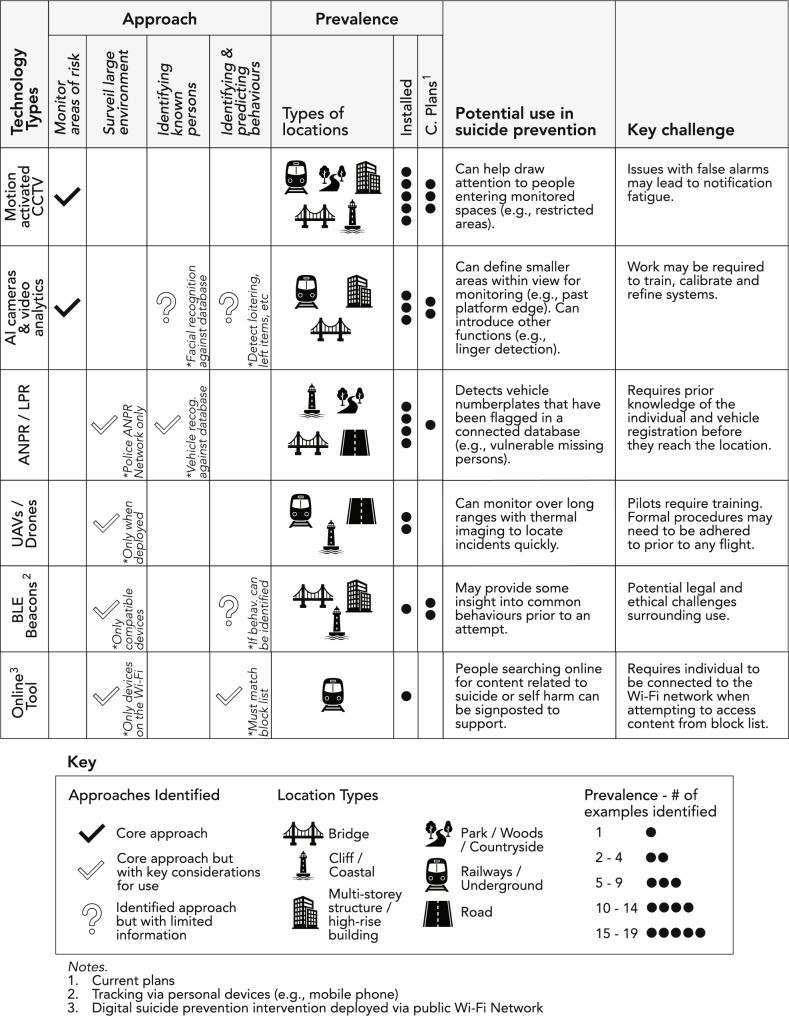
Summary of identified characteristics of smart surveillance technologies

**Fig. 2:**
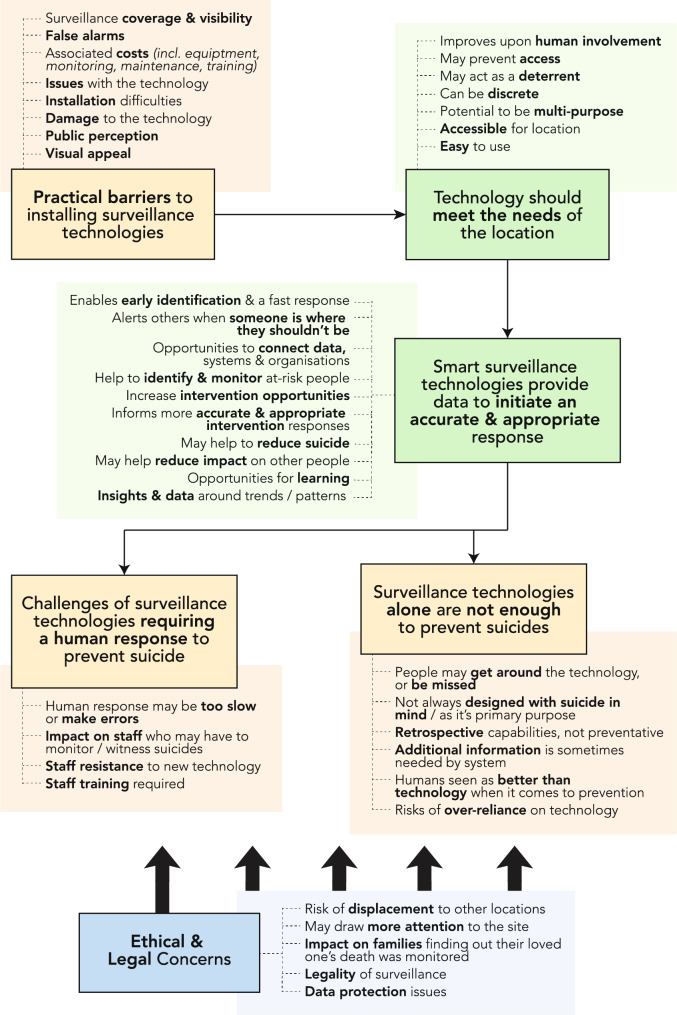
Content map of the qualitative findings

Visual summaries of the key findings from this survey can be found in Figs. 1 and 2.

Participants represented a range of sectors and organisations, including Local Authorities (34/108, 32%), Emergency Services (14/108, 13%) and the Health Service (11/108, 10%). Around 14% worked for organisations which managed transport infrastructure, including the rail industry (10/108, 9%). Others worked for organisations which oversee the management of green spaces (4/108, 4%) or properties (7/108, 7%). Both the technology (6/108, 6%) and security (4/108, 4%) industries were also represented in the survey. Participants worked across all regions of the United Kingdom (UK), with several working with multiple regions within their role (Table 2).

**Table 2:** *Summary of Participant Demographics*

	*N*	Example Location / Role
Region of United Kingdom		
England	88	
East of England / Anglia	12	*(Bridge*,* Multi-storey structure*,* Road)*
East Midlands	15	*(Railway)*
London	21	*(Bridge*,* Multi-storey structure*,* Railway*,* Green spaces*,* Road)*
North East	10	*(Bridge)*
North West	21	*(Bridge*,* Railway*,* Green spaces)*
South East ^a^	28	*(Cliff / coastal location*,* Multi-storey structure*,* Road)*
South West	11	*(Railway)*
West Midlands	15	*(Multi-storey structure*,* Railway)*
Yorkshire & the Humber	15	*(Bridge)*
Northern Ireland	6	*(Bridge)*
Scotland	15	*(Bridge*,* Railway*,* Road)*
Wales	9	*(Railway*,* Road)*
Sector / Industry		
Local Authority (e.g., Councils)	34	*Commissioner*,* Public Health*,* Mental Health*,* Suicide Prevention*,* Community Safety*,* Road Safety*,* Infrastructure*,* Parking Services*
Health Service (e.g., NHS Trusts, Health & Social Care Trusts, ICBs)	11	*Suicide prevention*,* Mental Health*,* Safeguarding*
Emergency Services (e.g., Police, Fire & Rescue)	14	*Suicide Prevention / Mental Health / Safeguarding*,* Real Time Suicide Surveillance*,* Community Safety*,* Designing Out Crime*
Rail Industry	10	*Suicide Prevention*,* Security*,* Route Performance*,* Research & Development*,* CCTV Manager*
Other infrastructure (e.g., Roads, Bridge Owners)	5	*Operations*,* Safety*,* Suicide Prevention*
Charity – Wellbeing / Mental Health	6	*Head of Service*,* CEO/Director*,* Programme Manager*
Charity – Nature / Wildlife	4	*Head of Nature Reserves*,* Facilities Manager*,* Project Manager*
Technology	6	*CEO / MD*,* CTO*,* Business Development*
Security	4	*CEO*,* CCTV Control Room Operative*,* Intelligence Manager*
Property Management (e.g., Shopping centres, car parks)	7	*Centre Management*,* Risk Management*,* Suicide Prevention*
Other	1	*Civil Engineer*

Note. Participants were asked to select all regions of the UK that they worked with in their role, with several working across multiple regions. Listed types of locations refer only to those which have had technology implemented or planned

^a^ Excluding London

Over half of respondents (57/108, 53%) indicated they were aware of at least one location where SSTs had been implemented. Twenty-two participants reported being aware of one or more current plans to install SSTs (22/85, 26%) and six participants also responded to questions about discontinued plans (6/74, 8%) Several locations also had multiple technologies implemented or projects planned. In total, 72 examples of SSTs being implemented or planned were identified (Table 3).

**Table 3:** *Summary of SST Projects Identified by Stage of Project*

	Implemented	Current plans	Discontinued plans
Awareness of SST projects ^a^	57 (53%)	22 (26%)	6 (8%)
Identified locations	55	20	6
Identified technology	48	16	8

Note. ^a^ Reflects the number and percentage of participants indicating “yes” to this question

### Location characteristics

Location type was provided for 55 locations which had implemented at least one SST project, 20 locations with at least one SST project currently planned, and six locations with discontinued plans for at least one SST project (Table 4). A quarter of these location types were bridges (20/81, 25%), primarily over water (15/20). 24% (19/81) were rail sites, the majority of which being stations (14/19). A further 21% of sites (17/81) were multi-storey structures (incl. high-rise buildings), for instance car parks (9/17) and commercial buildings (5/17). Other sites where technology had been implemented included roads (7/81, 9%), cliff and coastal locations (4/81, 5%), and green spaces i.e., parks, wooded areas, and the countryside (5/81, 6%).

**Table 4:** Location Characteristics for Identified Locations by Project Stage

	IT		CP		DP		Full sample	
	*n*	%	*n*	%	*n*	%	*n*	%
Type of Location	*N =* 55		*N =* 20		*N =* 6		*N =* 81	
Bridge	13	23.6	5	25.0	2	33.3	20	24.7
Cliff / Coastal Location	2	3.6	1	5.0	1	16.7	4	4.9
Multi-storey structure	13	23.6	4	20.0	0	0.0	17	21.0
Green spaces	2	3.6	3	15.0	0	0.0	5	6.2
Railways	13	23.6	4	20.0	2	33.3	19	23.5
Road	6	10.9	1	5.0	0	0.0	7	8.6
Other ^a^	6	10.9	2	10.0	1	16.7	9	11.1
“High-risk” location	*N =* 42		*N =* 14		*N =* 6		*N =* 62	
Yes	30	71.4	7	50.0	5	83.3	42	67.7
No	7	16.7	7	50.0	1	16.7	15	24.2
Unsure	5	11.9	0	0.0	0	0.0	5	8.1
Type of area	*N =* 42		*N =* 14		*N =* 6		*N =* 62	
Urban area ^b^	28	66.7	10	71.4	5	83.3	43	69.4
Rural area ^c^	4	9.5	2	14.3	1	16.7	7	11.3
Other	10	23.8	2	14.3	0	0.0	12	19.4
Access to site	*N =* 43		*N =* 14		*N =* 6		*N =* 63	
Public	35	83.3	11	78.6	5	83.3	51	81.0
Private	4	9.3	1	7.0	1	16.7	6	9.5
Other ^d^	3	7.0	2	14.3	0	0.0	5	7.9
Unsure	1	2.3	0	0.0	0	0.0	1	1.6
Uses for SSTs at location	*N =* 54		*N =* 22		*N =* 6		*N =* 82	
To prevent suicide / suicide attempts	36	66.7	15	68.1	5	83.3	56	68.3
To prevent accidental injury or death	16	29.6	9	40.9	1	16.7	26	31.7
To prevent trespass	26	48.2	12	54.5	1	16.7	39	47.6
To prevent crime / anti-social behaviour	36	66.7	18	81.8	3	50.0	57	69.5
Other ^e^	8	14.8	3	13.6	1	16.7	12	14.6

Note. Projects are categorised into *IT *Implemented Technology, *CP* Current Plans, *DP* Discontinued Plans

^a^ Primarily, participants used the “other” response to indicate multiple types of locations (including for major events and iconic sites). “Other” responses also notably included ports

^b^ Includes cities and towns

^c^ Includes the countryside and villages

^d^ Responses primarily environments with mixed access (i.e. public and private)

^e^ Includes crowd or traffic monitoring, object detection (e.g., weapons, hostile drones, revenue collection, and anti-terrorism)

Overall, around two-thirds of locations were known locally as being “high-risk” or “high-frequency” locations for suicide (42/62, 68%). At locations where SSTs had been installed, uses for the technology included preventing suicide / suicide attempts (36/57, 67%) as well as preventing crime and antisocial behaviour (36/57, 67%), trespass (26/57, 48%) or accidental injury and death (16/57, 30%). Suicide prevention was the 2nd most commonly envisioned use at locations with current plans (15/22, 68%) after prevention of crime and anti-social behaviour (18/22, 82%). This suggests that SSTs are generally used to support a range of use cases.

### Technology characteristics

A summary of all technological characteristics identified and the perceived effectiveness of said technology at preventing suicides can be found in Table 5. Notably, the majority of implemented SSTs were also reported as still being in use (38/41, 92%). Overall, participants gave a median score of 50.00 (IQR 18.75–70.75) out of 100 for perceived effectiveness of using the SSTs for suicide prevention. As might be expected, SSTs implemented or planned with suicide prevention as a primary use (12/70, 17%) were judged as more effective at preventing suicide and suicide attempts (Median = 74.00, IQR = 51.25, 80.00) compared to other projects (Median = 42.00, IQR = 12.50, 70.00; U = 153.50, *p* = .009).

**Table 5:** Summary of Technology Characteristics by Stage of Project

	Stage of Project (*N*)				Combined Sample			Perceived Effectiveness for Preventing Suicides		
	IT	CP	DP		*n*	%		Median	*N*	IQR
Type of technology	(*N* = 48)	(*N* = 16)	(*N* = 8)		(*N* = 72)					
ANPR	10	1	1		12	16.7		8.00	10	0.00–70.00
BLE Beacons	1	3	0		4	5.6		60.00	3	^a^
MA CCTV	17	6	2		25	34.7		55.00	21	20.00-72.50
AI camera / video analytics system	7	4	1		12	16.7		60.00	11	34.00–70.00
Drones	3	0	0		3	4.2		73.00	3	^b^
Virtual fencing	2	1	1		4	5.6		60.00	4	32.50-83.75
Other ^c^	8	1	3		12	16.7		42.00	10	14.50-84.25
Response activated	(*N =* 47)	(*N* = 14)	(*N* = 8)		(*N* = 69)					
Human response	36	13	6		55	79.7		55.00	49	31.50–77.50
Standalone intervention	6	1	1		8	11.6		62.50	6	37.50–88.50
Other ^d^	2	1	0		3	4.3		13.00	3	^e^
Unsure	7	0	2		9	13.0		50.00	7	30.00–70.00
Technology linked to	(*N* = 45)	(*N* = 14)	(*N* = 8)		(*N* = 67)					
Two-way audio system	7	1	1		9	13.4		80.00	8	50.00-87.50
One-way audio system	4	1	1		6	9.0		37.50	6	10.00–75.00
Audible warning / alarm	9	1	1		11	16.4		60.00	10	45.75–82.50
Visual warning / alarm	6	1	1		8	11.9		70.00	7	50.00–90.00
Call to emergency services	6	3	1		10	14.9		60.00	9	57.50–84.50
Control room alert	30	7	2		39	58.2		58.00	34	32.25–71.25
Text message to individual	3	1	1		5	7.5		45.00	5	31.50–55.00
Other ^f^	3	1	0		4	6.0		14.00	4	10.75–58.50
Unsure	10	3	5		18	26.9		42.00	16	12.00-68.75
Primary intended use for technology	(*N* = 47)	(*N* = 15)	(*N* = 8)		(*N* = 70)					
To prevent suicide / suicide attempts ^g^	9	2	1		12	17.1		74.00	12	51.25-80.00
To prevent accidental injury or death	17	8	5		30	42.9		60.00	26	42.00-81.25
To prevent trespass	16	4	1		21	30.0		56.00	19	34.00–80.00
To prevent crime / anti-social behaviour	27	8	4		39	55.7		53.00	34	17.50-70.75
Other	9	0	0		9	12.9		13.00	9	1.50–61.50

Note. Projects are categorised into *IT *Implemented Technology, *CP* Current Plans, *DP* Discontinued Plans

^a^ Perceived effectiveness of BLE Beacon ranged between 15–75

^b^ Perceived effectiveness of Drones ranged between 50–89

^c^ Includes radar, infrared sensors, online interceptive tools deployed over public WiFi networks, and technology to digitally observe electronic devices

^d^ Technology was piloted to develop use case (no response) or notify parking company

^e^ Perceived effectiveness of technology with “Other” response type ranged between 6–60

^f^ Includes a prompt on individual’s mobile phone (not text message), and a people counter

^g^ Category created based on free-text “Other” response

**Fig. 3:**
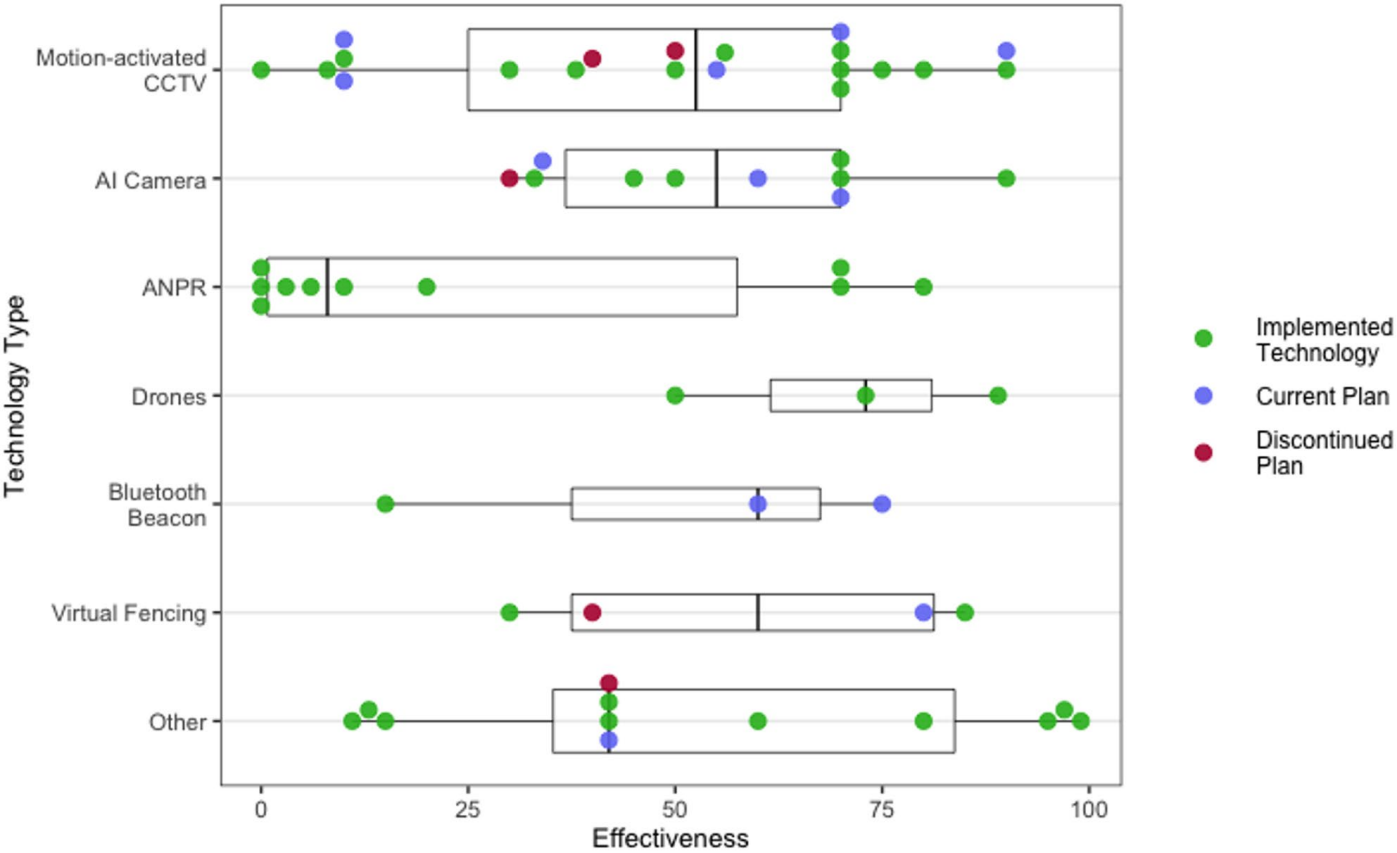
Perceived Effectiveness of Technology for Suicide Prevention by Technology Type. Note: Scatter points reflect responses across the three types of project stages, with boxplot representing median score and interquartile range. “Other” responses include radar, infrared sensors, online interceptive tools deployed over public WiFi networks, and technology to digitally observe electronic devices

The perceived effectiveness of SSTs for preventing suicides also varied within and across the different types of technology (Fig. 3). To understand why this may be the case, participant responses surrounding the use of “smart” cameras (e.g., using sensors or AI), ANPR, drones, and the monitor of personal devices are examined in the following sections (also see Fig. 1 for a visual summary). As well as these technologies, other SSTs identified as being used or planned across the locations included virtual fences, radar with analytics, and a sensor based system detecting when vehicles had stopped in moving traffic.

#### “Smart” cameras: motion sensors and artificial intelligence

Where identified, motion-activated CCTV and AI cameras / video analytics systems had all been implemented within the past five years. Across all locations, CCTV activated by movement or proximity (i.e., sensor based) was the most common technology to either have been installed (17/48, 35%) or included in current (6/16, 38%) and discontinued (2/8, 22%) plans. These cameras were implemented or planned across a range of locations, most commonly at Bridges (7/25, 28%) and Multi-storey structures (6/25, 24%). The perceived effectiveness of motion-activated CCTV varied considerably (Median = 55.00, IQR = 20.00, 72.50). Ratings were, however, significantly lower at the six sites which were not using the technology for suicide prevention (Median = 10.00, IQR = 7.50, 51.00) compared to the fifteen sites where the technology was being used for this purpose (Median = 70.00, IQR = 50.00, 75.00; U = 18.50, *p* < .05). As discussed later (see “surveillance technologies alone are not enough to prevent suicides”), this may in part be due to positioning of the cameras.

AI-based cameras and video analytics systems were identified at seven locations (7/48, 15%) and in one set of discontinued plans (1/8, 13%). However, a quarter of current plans did envision they would implement them in the future (4/16, 25%). Primarily, these technologies were deployed or planned on the Railways (5/12, 42%) and at Bridges (4/12, 33%). Perceived effectiveness for cameras and video analytics systems utilising AI also somewhat varied (Median = 60.00, IQR = 34.00, 70.00). AI cameras were deployed in a variety of ways. Similar to motion-activated CCTV, AI cameras are sometimes used for monitoring high-risk areas such as railways where cameras may be “positioned to alert staff to anyone going too close to platform edges and ends” (P40; AI Cameras) and used “perimeter analytics on platform to alert if breached onto line/lineside” (P25; AI Camera). The use of AI in this context was seen as being beneficial due to the support it provided for those responsible for monitoring multiple locations; “With over 8000 cameras, live detection is unlikely” (P25; AI Cameras).

The potential benefits of utilising AI when monitoring high-risk areas were also apparent when considering false alarms. False alarms were an issue for both motion-activated CCTV and AI cameras, but for different reasons. One site using motion-activated CCTV reported issues related to animals triggering false alarms causing “fatigue with notifications” (P90; MA CCTV). At another, an inability to set up “rules” for two different zones meant that it was not possible to also filter out incoming alerts from environments people were allowed to be in:

“2 x footpaths, 1 open, 1 closed this means the open footpath will constantly alert that there is individuals on the footpath, cannot manage this system to turn off the alarm on the open path and leave active on closed.” (P50; MA CCTV).

It may be that some of these issues could potentially be managed with more advanced technologies, such as AI cameras, but there was still a “Potential for high false alarm rate unless algorithm is correctly refined” (P42; Edge-based AI analytics). The work required in terms of “tweaking the system to lessen false alarms” (P106; AI Camera) were mentioned as a limitation or challenge for six projects implementing AI cameras or analytics systems. For instance, systems may require calibration to prevent commonly occurring features within the environment triggering false alerts:

“Two main issues have emerged. 1. The reduction of false activations, getting an effective patch on screen to pick up the most concern for welfare incidents without also picking up train movements and normal passenger activity.” (P40; AI Camera).

“Specification may need to be adjusted due to seasons. (Shadows, foliage and reflections may trigger false alarms)” (P25; AI Camera).

There were also at least four examples of AI cameras / systems being used or proposed to alert staff to other types of behaviour within environments such as at train stations:

“detect an amalgamation of activities from left luggage, trespass, weapons, and suicide prevention e.g., if a customer was at the loitering on a platform and several trains went by and the individual did not get on a train it would send an alert to the operator and in turn send a member of staff to check on the individual” (P77; AI Camera).

Additionally, the use of facial recognition was identified in one set of cancelled plans, but this was described as primarily being intended for crime prevention purposes.

Even seemingly unsuccessful AI Camera trials appeared to present a learning opportunity for future deployments: “Whilst the trials have been inconclusive in effectiveness we can use this to improve future projects.” (P40; AI Camera). While AI-based systems may require technical expertise and time, integrating AI into systems (including alongside smart sensors) could present opportunities to refine a system and potentially improve effectiveness. As one participant also highlighted, “Technology is evolving rapidly, remote battery 4 g cameras are now installed with AI and able to filter false alarms more effectively” (P8; MA CCTV).

#### ANPR

ANPR was identified as having been used the longest (“approximately 20 + years”) out of all the technologies. While ANPR cameras had been installed at ten locations (10/48, 21%), they were the focus of relatively fewer current (1/16, 6%) and discontinued (1/8, 13%) plans. As might be expected, ANPR was also primarily deployed or planned on roads (3/12, 25%) and at multi-storey carparks (3/12, 25%). However, there were also instances where it had been implemented at entry points and access roads for other types of sites. Overall, ANPR was judged to be the least effective technology at helping to prevent suicides (Median = 8.00, IQR = 0.00, 70.00). Indeed, participants highlighted that ANPR was only effective when a vehicle was involved, which was less common in some areas, and where there was knowledge of the vehicle’s registration details.

However, as illustrated in Fig. 3, three participants who had worked with the technology gave ANPR a score of 70 or higher. At lower scoring sites, ANPR tended to have been installed primarily for reasons such as revenue protection and to enforce road traffic laws, or were located in privately owned carparks. Conversely, sites where ANPR was judged as being more effective (i.e., 70 or higher) for suicide prevention were either connected directly to police control rooms or “alerts the emergency services immediately” (P12; ANPR). It may therefore be the case that ANPR cameras are most useful for suicide prevention when connected to the national system:

“Any vehicles which had information markers on them pertaining to a concern for welfare of the driver, which had been placed on the vehicle anywhere in the United Kingdom would alert the Port Police if this vehicle entered the Port” (P70; ANPR).

Yet, there were indications that ANPR may not always be readily associated as being a helpful tool for locating vulnerable individuals by emergency services, which may in turn impact its effectiveness at preventing suicides. While the system “requires a marker being placed on a vehicle to raise a concern for the driver/occupant” (P70; ANPR), one participant indicated “the Police will not always search the ANPR database immediately when searching for a vulnerable person” (P12; ANPR). Therefore, even ANPR cameras connected to national systems may not always be used to their full potential for suicide prevention.

#### Drones

Three locations were reported as having deployed drones (3/48, 6%). However, it should be noted that unlike the other technologies identified here, drones are often portable and therefore can be moved between locations. Indeed, one participant commented:

“Drones can be deployed almost anywhere… Drones are deployable at all kinds of location and transportable between incidents. Often deployed in high risk locations.” (P40; Drones).

The potential of drones helping prevent suicides was generally favourable (Median = 73.00, Range = 0–80). Qualitative data indicated that this favourable opinion stemmed from drones improving upon human capabilities due to having long ranges, thermal imaging and quick deployment meaning they can locate incidents quicker and easier than humans can. However, it was acknowledged that this does require training human operators to ensure the drones can be used effectively and appropriately. Also, they may be able to quickly locate and identify a vulnerable person, but nonetheless “this technology leads to a chain of provisions that could be too late when a person is intent on harming themselves” (P107; Drones).

#### Monitoring of personal devices

Several of the technologies identified would notably require a person in distress to be carrying a digital device (e.g., their mobile phone). For instance, movement tracking using BLE Beacons had been implemented at one location (1/48, 2%) but was planned at a further three sites (3/16, 6%). Key limitations around the use of personal device monitoring primarily related to the ethical challenges. On one hand it was acknowledged that these technologies would provide information about the behaviour of individuals prior to a suicide attempt which would help to “identify any common behaviours that can be used as markers for very high jumping risk” (P116; BLE beacons). Conversely, participants noted firstly that the technology “would rely on covert use of technology which may not be legally feasible” (P116; BLE beacons) and secondly “The covert nature of the surveillance and the reputational risk if this is discovered, which would then also highlight to bereaved relatives that their loved ones deaths were being monitored” (P116; BLE beacons). This highlights key ethical considerations around implementing this type of surveillance technology.

Another example was a digital suicide prevention intervention which appears in a users’ browser only when searching the internet using keywords related to suicide or self-harm. Rather than being a tool the user themselves implement, in this instance the tool was being deployed across a public Wi-Fi network. This pre-requisite for activation was noted as a limitation of this technology, in that “People need to be on the WiFi and accessing content on the block list” (P90; Online Interceptive Tool). Similarly, “at major stations, mobile data coverage is usually strong so the WiFi may not be used” (P90; Online Interceptive Tool). This means that vulnerable individuals may not be directed to support if they are not meeting the criteria for activation to occur.

### Activated response: technological & human-led processes

**Fig. 4:**
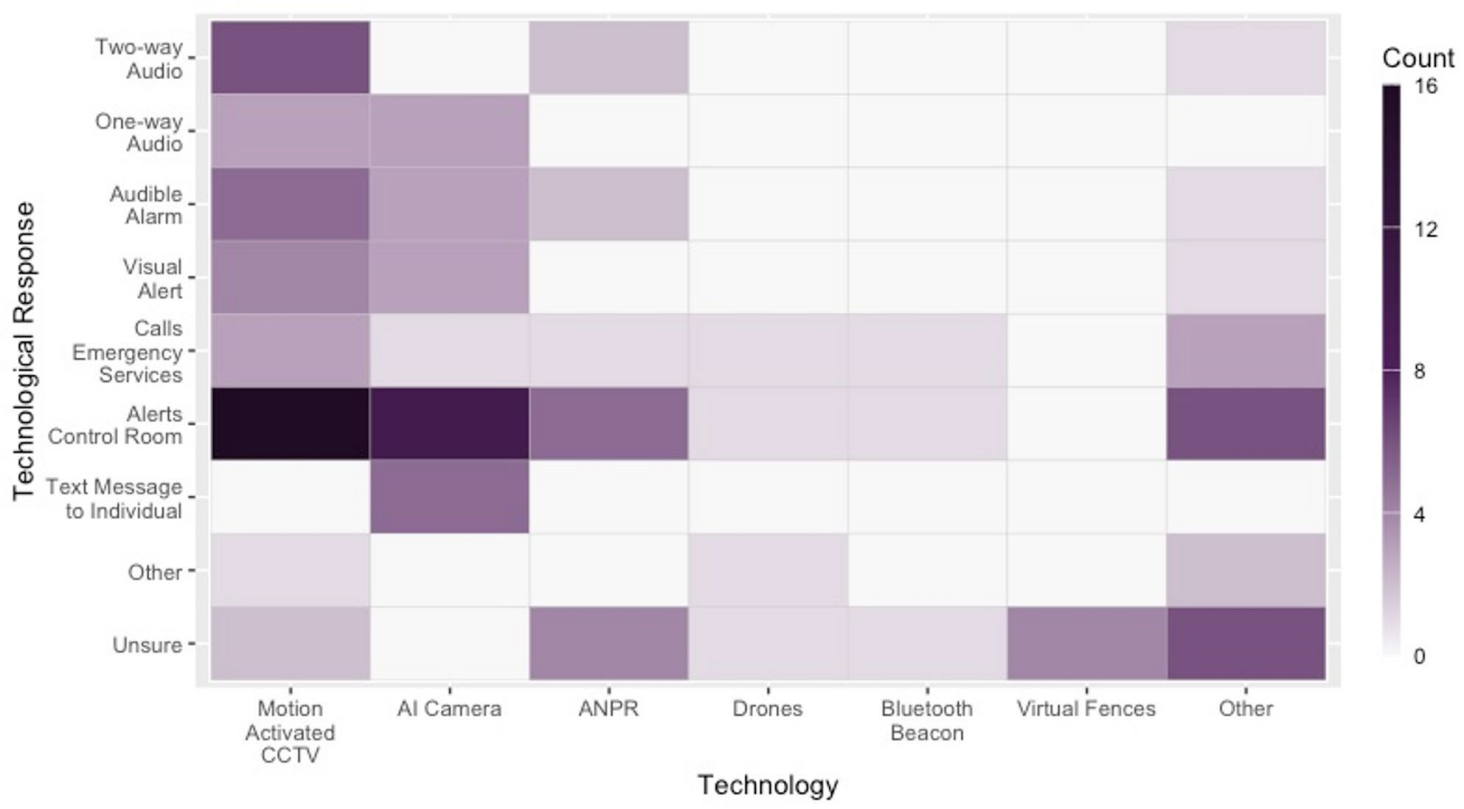
Technology Type by Response Activated Across All Project Stages. Note: Technology “Other” includes radar, infrared sensors, online interceptive tools deployed over public WiFi networks, and technology to digitally observe electronic devices. Technological Response “Other” includes an internal counter, and a digital prompt presented on the individual’s device

Figure 4 illustrates the relationships between deployed technology and activated response. Overall, SSTs were primarily designed to initiate a human response (55/69, 80%). Control rooms (39/67, 58%) were the primary means (actual or planned) of receiving an alert. On the whole, control rooms notifications were generated by Motion activated CCTV (16/39, 41%), AI cameras (10/39, 26%) and ANPR (5/39, 13%). Control room notifications were also the most common response for SSTs deployed or planned on the Railways (9/13, 70%), Roads (3/4, 75%), at Bridges (11/17, 65%) and at Multi-Storey Structures (7/13, 54%). Free-text responses indicated that, upon receiving a notification, control room staff may then “be required to assess the situation and make a decision on the best response” (P90; MA CCTV).

At some locations SSTs were then linked to one-way (6/67, 9%) and/or two-way (9/67, 13%) audio systems, allowing operators to communicate directly with those at the site. This approach was used across a range of location types, most commonly on the Railways (One-way; 3/6, 50%; Two-way; 3/9; 33%). Additionally, the three green spaces identified here notably reported only using (or intending to use only) two-way audio systems (2/3, 67%) or one-way audio and control room alerts (1/3, 33%). In some cases participants also described how staff may contact relevant teams to assist with a response at the location itself:

“Standardised process for alerting, Signalers (to implement live instruction/cautions to drivers in the vicinity [sic], BTP, Emergency Services and Control.” (P25; AI cameras)

“This Cameras are monitored both by [location] Police and our [location] Safer neighbourhood service where they can speak to the individuals through the camera as well as dispatch resources such as town centre wardens which may be more responsive/appropriate that [sic] police intervention. All staff have received Suicide Prevention Training.” (P13; MA CCTV).

Automated calls to emergency services (10/67, 15%) provided another potential means of initiating a human response. This was the most common response used or planned at cliff / coastal locations (3/6, 50%). Some AI cameras were also able to send text messages to individuals (e.g., pre-programmed phone numbers for managers or security teams), and this mode of response was most commonly found on the Railways (4/5, 80%).

Notably, relatively few examples were intended to operate as a standalone response (8/69, 12%). For instance, a trespass prevention pilot on the railways used AI cameras to issue a recorded deterrent message “You are nearing to the end of the platform and may be at risk of Trespassing” (P77; AI Camera). However, it is not clear how effective this type of system would be for preventing suicide specifically.

Qualitative analysis developed two themes related to use of technology which activated human-led responses. Both themes “Surveillance Technologies Provide Data to Initiate an Accurate and Appropriate Response” and “Challenges of Surveillance Technologies Requiring a Human Response”, are discussed in more detail below (a visual summary of all qualitative themes can be found in Fig. ).

#### Surveillance technologies provide data to initiate an accurate and appropriate response

Participants discussed the benefits of surveillance technologies for providing notifications and alerts that there may be a vulnerable person in a dangerous location. This was reported to facilitate opportunities for early intervention (n = 39):

“Enable a quick security response before the person can put themselves in danger” (P6; Virtual Fence).

Four responses indicated that having the data regarding the individual’s exact whereabouts and the capability for “real time observation” (P9; Technology to digitally observe electronic devices) also allows for a more targeted response by the appropriate services which could increase the chances of saving lives:

“If someone did jump, cameras could automatically locate and ‘lock on’ to the person in the water, enabling a better SAR response when every second is critical”. (P42; Edge-based AI analytics to detect anomalous behaviour).

Seven participants noted that technology may be particularly beneficial when the data is combined with that from other systems or organisations, for example linking ANPR data with reports of missing persons, or automatically sharing “data with other relevant public bodies” (P123; BLE Beacon). Further, eight responses suggested that collating this data across incidents would help to understand behavioural patterns that could help predict future incidents:

“It will identify any common behaviours that can be used as markers for very high jumping risk” (P116; BLE Beacon).

It could also help to inform ways of making high-risk locations safer in future planning:

“Intelligence to improve sites (Suicide Safer) Building in measures early with any new planning decisions to prevent issues at new sites” (P13; MA CCTV).

Through providing this information about individual incidents and understanding patterns of behaviour, four participants reported that they believe there will be a “reduction in attempts” (P130; MA CCTV) and that “this does result in a reduction in suicide” (P45; MA CCTV).

#### Challenges of surveillance technologies requiring a human response to prevent suicide

Overall, most technology (installed or planned) was intended to activate a human response (55/69, 80%). Whilst this can have the benefits outlined above of facilitating early and more targeted responses, challenges were also identified. It was stressed in 27 responses that the technology can only identify an incident and the responsibility still lies with staff to continually monitor the technology and to activate a response. This raised queries around what the most appropriate response would be and how to orchestrate this:

“Getting a proper, timely response plan in place for when activations occur. Smart Cameras can go in but who responds when they send an alert? This is harder to organise than cameras” (P40; AI Camera).

Participants also noted that the response “is open to human error” (P6; Virtual fence), in that individuals monitoring in a control room may miss alerts or may react too slowly. Moreover, it was acknowledged that even prompt responses to alerts may still be too late:

“The rapidity of any response would be insufficient to address a determined planned attempt” (P83; MA CCTV & AI camera).

It may be that the chance of human error is increased by the recurrence of false alarms, whereby technology will alert staff to an incident in error and the obstruction it has detected is in fact an animal or a leaf, for example. False alarms seemed to be quite prevalent, being noted as a challenge in 15 responses, and four people indicated that they were the greatest challenge to using the technology. This is concerning as participants reported “multiple false activations leading to apathy in response” (P40; AI cameras). Unfortunately, this could present a situation in which a genuine incident occurs but is overlooked because of false alarms, meaning a suicide is not prevented.

In addition to concerns for individuals at risk of suicide, three participants were also worried about the staff monitoring the technology and the possible impact on their wellbeing from witnessing a suicide. There were also concerns around staff’s capabilities using the technology, and the challenges of “getting enough people trained” (P40; Drones) and “staff resistance to use of new systems and equipment” (P40; AI camera). This highlights the importance of considering the needs of staff involved in using or monitoring the technology. Consequently, the technology being “easy to use” was endorsed as a key benefit. Similarly, technology that is “less reliant on people” (P14; MA CCTV) and is “automated to a certain extent” (P123; ANPR) was highlighted as beneficial, as it can overcome the challenges of the human response.

### Location-specific considerations

Three further themes were identified which relate to the environment SSTs would be deployed within (i.e., public spaces); “Technology Should Meet the Needs of a Location”, “Surveillance Technologies Alone Are Not Enough to Prevent Suicides” and “Barriers to Installation of Surveillance Technologies” (Fig. 2).

#### Technology should meet the needs of a location

Participants praised technology that was perceived as appropriate for their respective locations. This was true of both the design and the function. With regards to the design, technology being discrete was highlighted as beneficial in two locations as “it prevents knowledge of the site being a high-risk location” (P12; ANPR), which can be impactful in stopping the number of incidents from increasing. For implemented projects, around half of locations had technology that was visible (23/47, 49%) or advertised (24/51, 49%) to site visitors. Conversely, technology that was more explicit was valued by four participants for acting as a deterrent, for example it being “very loud to deter access” (P8; MA CCTV) or acting as a “visual deterrent” (P126; virtual fence), which can also reduce the number of incidents. The accessibility of the technology in relation to the infrastructure at different locations was also discussed:

“The technology is accessible for a remote coastal site with limited infrastructure / reception” (P12; ANPR).

Given the complexity of some locations, the multi-purpose functions of technology were noted as one of the main benefits by three participants. The technologies also played a role in “crowd management” (P39; MA CCTV), “pedestrian safety” (P39; MA CCTV), and “crime prevention” (P123; ANPR). However, some use cases may be perceived as being more effective at preventing suicide. For instance, SSTs primarily used to prevent accidental injury or death were judged as being more effective at preventing suicide and suicide attempts (Median = 60.00, IQR = 42.00, 81.25) compared to those not primarily used to prevent accidental injury or death (Median = 33.50, IQR = 10.00, 70.00; U = 265.00, *p* = .004). This was not the case for SSTs with a primary use cases of either trespass or crime prevention (Additional file 4). As such, perceived effectiveness may also be greater when technology is implemented with specific safety aims in mind.

Finally, the technology having capabilities beyond that of a human was noted as advantageous by nine participants. For example, in larger locations drones can be particularly beneficial as they can move quicker and cover more area than foot patrols, and they “have thermal imaging capability and a long range” (P40; Drones).

#### Surveillance technologies alone are not enough to prevent suicides

Although the benefits of surveillance technologies were widely discussed, a theme within the data was that they alone are not enough to prevent suicides. It is outlined above how a human response was required for the majority of technologies discussed. Indeed, while there were several examples of technology operating as a standalone response (8/69, 11.6%), only two (3%) did this exclusively (i.e. no human response). In these instances, participants argued that the technology “doesn’t physically prevent anything happening” (P53; ANPR) and “is exclusively a retrospective rather than a proactive measure” (P83; MA CCTV; AI camera).

An additional challenge discussed by 12 participants was that people may avoid detection if they are aware of the technology and how to bypass it, or they may not meet threshold for detection. For example, someone may only be detected via ANPR if they are actively being looked for, i.e., they have been reported as a missing person. Similarly, surveillance via the internet may not be activated if the individual is not connected to the relevant public Wi-Fi network:

“At major stations, mobile data coverage is usually strong so the WiFi may not be used” (P90; Online interceptive tool).

According to 16 participants, the coverage of the technology can also mean that individuals go undetected, for example on the railways it is “impossible to cover all the tracks” (P59; Video Analytics, AI object classification, Radar). This is also true of other locations, such as ports:

“Not all areas where persons in crisis can access dangerous locations are covered by motion detecting cameras” (P70; MA CCTV).

Participants also discussed how the visibility of cameras may be inadequate, particularly in poor weather conditions or at nighttime:

“May not work as well in dark conditions unless there is enough of a thermal imagery dataset to train the AI Early notification of persons in distress enabling rapid response and intervention” (P42; Edge-based AI analytics to detect anomalous behaviour).

In response to the above points, seven participants felt that more information is needed in order for the technology to function effectively. For example, ANPR technology cannot work without “knowledge of the individual’s vehicle” (P123; ANPR) or BLE Beacons are “no help if there is no record of the vulnerable person on the database” (P123; BLE Beacons).

It was also noted that one technology alone is not sufficient for suicide prevention:

“One system alone will not resolve the issue, but other measures are being considered to make the site safer” (P126; BLE Beacons).

Subsequently, it is encouraging that the majority of the sites where technology had already been implemented were also utilising other suicide prevention measures including signage (29/37, 78%), patrols (23/37, 62%), barriers (18/37, 49%) and staff / gatekeeper training (14/37, 38%).

Another reason that technology alone may not be adequate to prevent suicides is if the technology has not been implemented primarily to detect suicide attempts, or even with suicide prevention in mind. Indeed, while a majority of sites were using or intended to use technology for suicide prevention purposes (56/82, 68%), less than a fifth of projects actually identified suicide prevention being a primary use (12/70, 17%). Technology was more commonly used primarily to prevent crime and antisocial behaviour (39/70, 56%), accidental injury or death (30/70, 42.9%), or trespass (21/70, 30%). This may mean that the technology (and any corresponding systems and processes) is optimised to detect and prevent other types of prioritised events:

“It is not the principle purpose of the surveillance, which is to detect intrusion. There is a certain applicable benefit that coincides with suicide risk mitigation, but the principle purpose has to be prioritised as it is a paid for service that has been customer specified” (P83; MA CCTV & AI Camera).

#### Barriers to installation of surveillance technologies

Practical considerations regarding installation difficulties were also identified as barriers to the technologies. The cost of installation and maintenance was raised by eight participants, three of which cited costs as the greatest challenge facing technology use. Where disclosed (*N* = 21), the median estimated cost for implementing technology was £25,000 (IQR £6,229 - £30,000). This ranged from £350 for motion-activated CCTV / AI cameras to £125,000 for an edge-based AI analytics system to detect “anomalous behaviour”. Seven participants also provided estimations for ongoing costs. This started from £13 per month covering a camera’s sim card, up to monitoring costs of £30,000. In addition to monitoring costs, one participant noted an annual cost of £2300 for software licensing and support.

Notably, source of funding also varied across the sites. Ten of the projects mentioned funding from local authorities (including ringfenced public health funding for suicide prevention), while two projects were to be funded by devolved governments. Several projects were identified as having public sector organisations as funding sources (both potential and actual), primarily where they managed the infrastructure (nine projects). Additionally, thirteen projects were indicated as being privately funded, however, this was primarily for privately owned locations with levels of public access such as multistorey carparks, commercial buildings and ports. Therefore, the possible routes for generating additional funds to install SSTs, and the priorities of those who go on to fund it, will differ across location.

There were also installation barriers specific to different locations, including being far away from power sources and “some locations are extremely expensive to make changes to due to engineering complexities” (P13; MA CCTV). Notably, SSTs were primarily implemented or planned exclusively for urban areas (43/62, 70%). However, it is important to acknowledge that the principles of urban planning do not necessarily apply to more rural or remote locations. Indeed, remote locations were noted as having more nuanced issues that should be considered:

“Coastal and remote countryside sites are often located far away from power infrastructure and have poor mobile phone reception. They are often much larger in scale and can be many miles long. Care should be taken to separate such sites from urban ‘built structures’ and not transfer conclusions” (P12).

Finally, other practical barriers to installation included health and safety issues, the technology being “bulky” (P77; AI Camera), and being an “unattractive measure, though this may be enhanced using something more aesthetically pleasing over time” (P126; Virtual fence). The visibility of the technology was also an issue due to the risk of vandalism or damage:

“It works well but has been subject to minor vandalism” (P12; ANPR).

### Understanding effectiveness

Many of the projects (21/37, 57%) had evaluated the impact of the technology, or had formal plans to do so. Types of evaluations identified included making comparisons against historical data or evaluating performance against other implemented technologies. Some identified evaluation processes included the use of experts such as data analysts, academic partners, and specialist companies for AI testing and validation. It is not clear, however, how much suicide prevention formed part of these evaluations.

There appeared to be no plans to evaluate impact for at least 30% of projects (11/37). Furthermore, one participant noted for a project there was “No formal evaluation yet but a lot of anecdotal evidence is available” (P40; AI Cameras). Yet, elsewhere, there were indications that relying on informal evidence could create challenges in the future:

“Maintenance budget cancelled approx 5 years ago due to lack of business case evidence of effectiveness. But was successful in deterring many trespass events but not quantified by project manager” (P8; MA CCTV).

Participants also highlighted that formally evaluating the effectiveness of SSTs in regard to suicide prevention was not always a straightforward process. For instance, the ability to isolate effects in the data were raised as potential barriers to formal evaluation:

“We are not sure how to evaluate, was implemented as part of a series of suicide prevention methods e.g. nightime closures of the footpaths, opening the hub, additional CCTV” (P50; MA CCTV).

“Very effective system in deterring trespass, difficult to ascertain who was trespass, thief or suicidal. So hard to build statistics for suicide prevention” (P8; MA CCTV).

### Ethical & legal concerns

In addition to the practical challenges already discussed, participants also raised specific ethical concerns (Fig. 2). This included legal issues around the “covert use of technology” (P116; BLE Beacons) and the implications of this for human rights:

“Although I am of course keen to prevent suicides, I am very concerned about the use of these technologies generally, as well as for this purpose. I think this raises many concerns about human rights, freedoms, and much more.” (P101; Additional Comments).

In particular, two responses suggested there were key challenges around the “public perceptions of being monitored” (P116; MA CCTV) as well as the impact on families:

“The covert nature of the surveillance and the reputational risk if this is discovered, which would then also highlight to bereaved relatives that their loved ones deaths were being monitored” (P116; BLE Beacons).

The actual legalities of deploying these technologies appeared to be unclear, however. Indeed, one participant noted that misunderstanding of the law was one of the biggest challenges for deploying SSTs in public spaces.

Furthermore, there were also worries that the technology could in fact exacerbate suicide at different locations by drawing attention to the site, which could “increase notoriety of location as a suicide opportunity” (P130; MA CCTV). Conversely, three participants worried that the technology “could potentially ‘displace’ suicide attempts to another location if technology becomes widely known” (P42; Edge-based AI analytics & AI cameras). This raises ethical concerns around the possibility of technology causing harm.

Finally, three participants discussed the risk of creating an over-reliance on technology in place of human contact. This was deemed to be an issue due to the belief that “humans are always better than technology when it comes to preventing suicides” (P51; Various SSTs). One specific challenge discussed was the possible bias with technology assessing suicide risk:

“Overall when using systems such as AI to support in the prevention of suicide we still have to understand that AI is not perfect. There is also the issue of bias when creating a programme to single out groups of people in public spaces in this case those who are experiencing suicidal thoughts. How does the programme decide who does or does not fit in that category and what steps are then taken to prevent the immediate risk” (P79; Additional Comments).

## Discussion

Professional stakeholders working with public locations across the United Kingdom were surveyed about the implementation and plans for using smart surveillance technologies, particularly in the context of suicide prevention. Seventy-two examples of SSTs were identified across a variety of location types (including bridges, the railways, and multi-story structures). Implemented and planned technologies included ANPR, Motion-activated CCTV, BLE Beacon technology, Drones and cameras or systems utilising AI-based video analytics. Most of the implemented systems sent alerts to control rooms and in a number of instances, provided a means of speaking directly with a person at the location via two-way audio. Relatively few systems operated as standalone interventions.

Many of the identified locations where SSTs were implemented or planned were “high-risk areas”. The most commonly reported SSTs were CCTV cameras activated by movement or proximity; the majority of which were deployed at high-risk areas. These systems were generally judged as being effective for suicide prevention when used for that purpose. Participants generally noted that these sensor-based systems helped reduce reliance on people to monitor an area. However, issues related to false alarms were apparent. While motion-activated CCTV may not be appropriate for use in areas with high foot traffic, the present work suggests integrating both sensors and AI into surveillance systems may be a helpful way of reducing false alarms in less busy environments.

There were also several examples of AI-based video analytics being deployed through cameras or edge-based systems. A number of these sought to identify individuals who crossed into restricted areas (e.g., lineside on the railway). The potential ability to locate and “lock on” to a person in water was also highlighted as something that could aid rescuers, and would arguably not be technologically possible without the use of computer vision processes. However, such systems were again not without issues, as false alarms could be triggered by factors such as train movements and reflections. Yet, the ability to adjust specifications, apply previous learnings, and the rapid evolution of these technologies were seen as potential benefits for using AI-based systems to monitor high-risk areas.

Additionally, there were a small number of examples where AI-based systems were being used or proposed to detect “behaviours”. However, these were notably focused on specific actions such as lingering, left objects, and crowd analytics, or the ability to identify specific objects (e.g., weapons). Previous research suggests that lingering [53] and leaving objects [54] may be actions that could act as markers of potential suicide attempts in rail settings. As one participant here noted, by helping identify these actions and issue relevant alerts, integrating AI into surveillance systems can help to inform staff when to check on potentially vulnerable individuals. However, from an ethical perspective, participants raised concerns regarding the use of SSTs to infer suicide risk, particularly the potential for bias or over-reliance.

Several participants expressed that the use of camera and sensor-based systems may be limited by the size of area they can cover. While their use may be appropriate for enclosed or smaller environments, “public spaces” may also include vast stretches of outdoor space. As illustrated here, the use of drones may be helpful for supporting human-led monitoring of a larger environment. However, the processes around deployment and need for training were also identified as limitations. The implementation and proposed use of BLE Beacon technology was also identified. This was viewed as potentially being useful for better understanding behaviours prior to a suicide attempt but also presented legal and ethical challenges. Overall, the use of SSTs for the surveillance of larger areas was less established and prevelant compared to other approaches.

Another identified benefit of deploying SSTs was their ability to provide data that could help inform rescue responses. Firstly, it was suggested SSTs could help identify vulnerable individuals before they were in a position of danger. As users may be responsible for carrying out a range of tasks or monitoring a number of locations, the ability for alerts to draw attention to a potential incident may be one way in which SSTs can support suicide prevention efforts. The ability to identify and track the whereabouts of an individual, as well as use data to inform future decisions, were also raised as perceived benefits. Several participants felt the data produced by SSTs were helpful for preventing suicides. These beliefs are supported by previous research from Lee et al. [26], who found installing and optimising sensors on a bridge in Seoul increased the number of rescue events on the bridge and in water. Therefore, the data that SSTs produce when monitoring areas could help to automate and optimise aspects of the response process.

While several participants identified the ability for technology to connect to or share data with other relevant public bodies as being a benefit, there were also a number of challenges identified regarding the practicalities of using SSTs to detect known, vulnerable persons. Firstly, this feature may only prove useful if the system is integrated with a relevant network. This may therefore present barriers for some private organisations managing public spaces. Moreover, the findings suggest that for certain systems to prove useful in preventing suicide, the databases they connect to must contain relevant and accurate information (e.g., markers placed on a vehicle, keywords). For instance, here it was noted that ANPR may be effective at alerting authorities when a marked vehicle is detected entering an area, but another participant highlighted that vulnerable people were not always searched for on the system immediately. Similarly, an online interceptive tool which responded to searches made via public Wi-Fi described here relied on individuals using specific keywords from a blocklist to activate the intervention. Ensuring blocklists remain up to date with known content evasion techniques (e.g., word camouflage) is therefore vital. As the responsibility for creating and maintaining these types of datasets may often lie with another organisation or team, such approaches may therefore require effective cross-party collaboration to optimise formal processes around use and database management.

The need for humans to physically respond to alerts was also highlighted by participants. Several potential challenges were raised around this. Firstly, what actually happens if an alert is raised; where does responsibility to monitor a system and provide a physical response sit. Moreover, would any such response be quick enough to intervene. Indeed, Silla [38] previously noted that SSTs may prove more effective at preventing suicide in areas where a rapid response is more feasible (e.g., urban areas). In the future, it may be that AI-based... AI-based solutions could help to inform and optimise emergency response coordination. However, as highlighted here and in previous research [59, 61], constraints around organisation-level issues such as budget, time, resourcing, and training can also dictate the feasibility of providing a physical response.

As well as any on-the-ground response, the potential impact surveillance systems may have on the humans tasked with monitoring them (e.g., on wellbeing after witnessing a suicide) was also raised. Notably, UK rail industry guidance on responding to potentially traumatic events acknowledge the potential impact on those who encounter aversive details of such events, including those monitoring camera footage [62]. They also highlight the importance of implementing and auditing simple, structured processes to support employees following a traumatic event. Said guidance may notably prove beneficial for other industries, including where monitoring by a third-party occurs (e.g., an external security company).Given some AI-based systems may also require those with technical expertise to encounter footage for model training and evaluation, any future guidance and processes should factor in these individuals also.

Participants also noted concern regarding the attitudes of staff towards using SSTs. Here, it was suggested that new systems may face resistance from some staff. This adds to previous research which suggests staff may be concerned about SSTs increasing workload or potentially replacing them entirely [59]. It is also thought that factors such as attitudes towards automation, general workload, cognitive overhead (tasks associated with using the automated system itself), trust in a system, self-confidence and perceived risk can influence decisions around using SSTs [63]. As the present research suggests human involvement is perceived to be vital in suicide prevention work, effective engagement and communication with the staff who are actually working with SSTs day-to-day should be prioritised to help address such concerns. Of particular note here were concerns raised around the impact of false alarms on staff. As previously discussed, false alarms appeared to be relatively common and difficult to resolve entirely. However, their impact on the individuals working with SSTs should also not be underestimated, as research suggests higher false alarm rates may lead to reduced response times or even system disuse [63]. Indeed, one previous trial of a sensor-based trespass detection system ended after the local police department monitoring the system muted all alerts and indicated they would continue to do so even if said system was refined further [58]. As such, working closely with operators to come to agreements on “acceptable rates” of false alarms may be important to help prevent SST disuse over time.

One of the key themes identified was the requirement for technology to meet the needs of a location; for instance, by supporting multiple use cases (e.g., trespass prevention). While the majority of SSTs discussed here were being used for suicide prevention work, suicide prevention was often not the primary purpose for installation. Deploying SSTs with multiple use cases in mind was seen as both a benefit and also a limitation. Arguably, multipurpose SSTs may allow costs to be shared or support suicide prevention efforts when there is no dedicated funding. However, as one participant noted, the primary use case will likely be prioritised. As illustrated here, the effectiveness of using SSTs for suicide prevention may be greater when the system has been developed and installed with that purpose in mind. Given the range of functionalities that may be added to AI video analytics systems at installation or over time, organisations deploying SSTs primarily for other reasons (e.g., trespass prevention, crime prevention) may still wish to involve colleagues or experts involved in suicide prevention to optimise performance. However, it was also noted by participants that shared use cases could present issues in the context of evaluation. For instance, where an SST can detect “trespass” it may not be possible to distinguish between a vulnerable trespasser and those trespassing for other reasons. While alerting staff to trespass may not present an issue in terms of “system performance” (as regardless of intent, trespass may pose a safety or security issue), participants highlighted that this could present challenges when it comes to evaluating in the context of suicide prevention specifically. As inability to demonstrate effectiveness was also identified as a reason for funding to be discontinued, more work is needed to support organisations on how to best evaluate SSTs in the context of suicide prevention.

The present research also highlights the importance of testing SSTs in live, real-world settings when seeking to understand whether they may be helpful for preventing suicides in public spaces. Indeed, as illustrated here, these complex environments can present a range of specific issues that may impact implementation. For instance, participants indicated that poor weather conditions, time of day, change of seasons, vehicle movements, and vegetation growth could affect the performance of an SST. Previous research has also reported issues relating to power fluctuations [58] and objects flying in the wind [26]. Such practical issues should also be considered when identifying and planning future installations. Yet notably, as one of the themes in the present study identified, the use of SSTs alone may also not be enough to prevent suicides. Participants reported that individuals may find ways to bypass technology if they are aware of it. This may be a particular issue when areas are not covered by the SST (another issue identified by participants): reviewing placement of any cameras or sensors after deployment at high-risk locations may therefore be beneficial for ensuring sufficient coverage (see [26]). However, even then, participants suggested that there may be situations where individuals do not meet the threshold for detection. As such, reliance on SSTs alone may mean potentially vulnerable individuals are not always identified.

Another way in which this theme was demonstrated was through the need for other measures to be present. Participants noted that simply installing an SST does not resolve the issue of suicide at a location, and that SSTs could not physically prevent anything from happening. Many of the sites had other measures in place, including signage, patrols and barriers. Previous work suggests that combining SSTs with measures such as barriers may be useful to provide additional time to issue a response [14]. Yet, as highlighted here and by others [33] isolating effects attributed to an SST specifically can present further challenges for organisations needing to understand effectiveness.

Several barriers to installing SSTs in public spaces were identified. Firstly, were practical issues such as legality, costs and source of funding. Notably, as the implementation of such systems can often be controversial, the findings here highlight a need for greater clarity around the legalities of deploying SSTs. However, the costs of installing technology were identified as being one of the greatest barriers to using the technology. Ongoing costs related to monitoring, as well as licensing and support must also be considered. Who then funds this technology varies across sites (e.g., location type, ownership). This could have important implications for which use case is prioritised, which as illustrated here, could influence effectiveness for helping to prevent suicides.

While previous work has identified that availability of human responders may be an issue for implementing SSTs in rural areas [38], it was also apparent here that disparities in digital infrastructure (e.g., digital connectivity, electricity supply) may present specific challenges for these locations. For example, it may not be feasible to deploy AI-based surveillance systems requiring high network bandwidth at certain locations where digital infrastructure improvements are required (something that may go beyond the control and resources available to organisations seeking to implement them for suicide prevention reasons). Even in instances where these SSTs “work”, if the digital infrastructure around the site is insufficient there may be a risk of impairing communication networks for local communities [58]. This may not only be inconvenient for those impacted, but also risks amplify any exisiting digital inequalities that may indirectly affect health. Indeed, previous work suggests access to high-speed, broadband internet can help reduce poverty and unemployment locally, but is also associated with improved mental and physical health in the local community, including reductions in the number of deaths by suicide [64]. Similarly, recent work suggests increasing inequalities in digital infrastructure may have a negative impact on the mental health of those living in rural areas [65, 66]. These examples highlight why factors such as digital infrastructure requirements are important considerations when thinking about deploying SSTs for suicide prevention purposes, particularly in rural areas.

Interestingly, local digital infrastructure may influence some people’s digital behaviours when they are situated within public spaces, which may then impact the effectiveness of certain SSTs. One SST identified required users to connect their personal devices to the public Wi-Fi network, so that any searches could be monitored for specific keywords which would then trigger a digital suicide prevention intervention. As participants identified here, use of public Wi-Fi may be less common where mobile data coverage is stronger (e.g., at major stations). However, in some areas of the UK, higher levels of digital inequalities may arguably increase community reliance on free, public Wi-Fi networks. For instance, individuals in deep rural areas may be more reliant on fixed internet connection points (e.g., at home, work, or in public spaces) due to poorer mobile coverage [67]. While further research is still needed to gauge whether these digital interventions are indeed effective at helping to prevent suicides generally, understanding where people may be more likely to be connecting to public Wi-Fi due to necessity (e.g., areas with higher digital inequalities) may also be beneficial. 

### Strengths & Limitations

To our knowledge, this is the first study to scope which SSTs are being used in practice and/or considered for suicide prevention. The mixed-methods approach used here also allowed for trends in the use of SSTs to be identified and better understood within the context of public spaces and suicide prevention. Through gathering feedback on a variety of smart technologies (including AI-based systems), it is hoped the findings presented here can help support decisions around implementing and optimising “smart” surveillance systems for suicide prevention purposes both now and in the future. While previous research in this area has also focused primarily on a specific type of location (e.g., the railways), by recruiting professional stakeholders who work across a range of public location types, the present work offers insights which may support cross-location learning.

However, it should be noted that the findings presented here represent only one perspective of SSTs being used for suicide prevention in public spaces. Specifically, those implementing or working with them. Given the ethical and privacy concerns surrounding the use of these technologies, more research is required to understand the perspectives of the individuals who may be on the receiving end of these technologies.

Whether SSTs emitted audible and/or visual alerts at the location or towards staff (e.g., in a control room) was also unclear here. There is an increasing need to understand whether entirely “autonomous” systems would be effective at preventing suicide. While real-time sound warnings have previously been identified as potentially promising for preventing suicides [38], trespass prevention research indicates that repeat preventers may become desensitized to audible deterrents over time [33]. If the effectiveness of such a system is context specific, autonomous systems may not be helpful when an individual’s motivation to harm themselves is stronger. Future research is therefore also required to understand whether systems issuing only audible deterrents and/or visual alerts are in any way effective at preventing suicide.

## Conclusion

Professional stakeholders were surveyed to understand the range of SSTs currently being used across the UK and the practicalities of implementing them in public spaces for suicide prevention. The majority of SSTs were implemented to monitor high-risk or restricted areas, using AI and / or smart sensors to do so. Relatively fewer examples were used to surveil larger areas, or utilised AI to detect specific behaviours. Moreover, while the use of ANPR appeared to be more common, very rarely was it used for suicide prevention purposes. Yet, in specific contexts, ANPR’s ability to assist in locating known, vulnerable persons was seen to be helpful.

The ability for SSTs to provide data to help guide rescue responses was identified as a potential benefit, either during a real-time attempt or to inform future decisions. The importance of ensuring SSTs met the needs of a location was also raised, as “suicide prevention” may be one of several use cases for systems monitoring public spaces. Participants also generally felt that a human response was still required when using SSTs, but also noted the challenges with providing this in regard to determining where responsibility for this lay, the feasibility of issuing a rapid response and the impact on those whose job role would be affected by the SST. A number of barriers to installing SSTs in public spaces were also identified, including costs, funding, and existing digital infrastructure. Around a third of the projects had no plans for evaluating the impact of the SST, but the difficulties around determining how best to approach this was also noted. Several ethical concerns were raised, particularly around the legalities of deployment and the potential for inadvertently causing harm. Finally, several participants noted that SSTs alone could not physically prevent suicides, and that other measures are also required. Together, the findings provide key insights into the practicalities of implementing and using AI-supported surveillance and other smart technologies for suicide prevention in public spaces. 

## Supplementary Information

Supplementary Material 1.

Supplementary Material 2.

Supplementary Material 3.

Supplementary Material 4.

## Data Availability

The datasets generated and analysed during the current study are not publicly available due to their sensitive nature but redacted, fully anonymised data are available from the corresponding author on reasonable request.

## Abbreviations

AI: Artificial Intelligence
ANPR: Automated Number Place Recognition (also known as LPR)
BLE: Bluetooth Low Energy
CCTV: Closed-Circuit Television
LFR: Live Facial Recognition
MA CCTV: Motion Activated CCTV
NSPA: National Suicide Prevention Alliance
SST: Smart Surveillance Technology
UAV: Unmanned Arial Vehicles (e.g., Drones)
UK: United Kingdom
