# Supplementary material for: The use of smart surveillance technologies for suicide prevention in public spaces: a professional stakeholder survey from the United Kingdom
Source: BMC Public Health. 2026 Mar 19;26:1382. doi: 10.1186/s12889-026-26739-0 (PMC13123109; doi:10.1186/s12889-026-26739-0)
Supplement: Supplementary file 1 — Supplementary Material 1. [file 12889_2026_26739_MOESM1_ESM.docx]

**Appendix D**

Perceived effectiveness for suicide prevention by primary use case

**Table D1**

*Summary of perceived effectiveness of SST for preventing suicides by primary intended use*

|  | Yes | | N/A | |  |  |
| --- | --- | --- | --- | --- | --- | --- |
| Primary intended use for technology | *N* | Median (IQR) | *N* | Median (IQR) | *U* | *p* |
| Prevent Accidental Injury or Death | 26 | 60.00 (36.80) | 36 | 33.50 (60.00) | 265.00 | .004 |
| Prevent Trespass | 19 | 56.00 (40.50) | 43 | 42.00 (58.00) | 309.00 | .128 |
| Prevent Crime / Antisocial Behaviour | 28 | 53.00 (46.50) | 34 | 42.50 (45.00) | 427.00 | .492 |
| Prevent Suicides / Suicide Attempts^a^ | 12 | 74.00 (16.30) | 50 | 42.00 (56.50) | 154.00 | .009 |

*Note*. Participants were able to select multiple options for primary intended use.

^a^ Category created based on free-text “Other” response.
