# Supplementary material for: The use of smart surveillance technologies for suicide prevention in public spaces: a professional stakeholder survey from the United Kingdom
Source: BMC Public Health. 2026 Mar 19;26:1382. doi: 10.1186/s12889-026-26739-0 (PMC13123109; doi:10.1186/s12889-026-26739-0)
Supplement: Supplementary file 2 — Supplementary Material 2. [file 12889_2026_26739_MOESM2_ESM.docx]

**Appendix C**

Overview of Data Cleaning

**De-identifying analysis dataset**

Variables containing identifiable data (e.g. IP address, contact details, organisation name, role) were removed from the main dataset. Additionally, where free-text responses contained identifiable information, this was redacted.

**Removed responses**

It was pre-agreed by the team that participants who did not answer any questions following demographic questions would be removed from analysis (e.g. regions of UK worked with) as no relevant information for the study was provided. In total, 21 participants were removed for this reason. Furthermore, responses about two sets of discontinued plans were removed as the technologies discussed were beyond the scope of the present research (e.g. social media monitoring, real-time surveillance).

**Edited responses**

***Recoded “Other” responses***

Where “Other” responses reflected multiple choice options, these were recoded to better reflect the descriptions provided. Affected variables and key examples are provided in Table C1.

**Table C1**

*“Other” Location Description Examples and Updated Codes*

| “Other” description provided | Recode | Recode - Subcategory |
| --- | --- | --- |
| Location type |  |  |
| “Shopping centre & multi-storey carpark” | 3 – Multi-storey structure / high-rise building | 3 - Commercial |
| “Bridge” | 1 - Bridge | 1 – Over water (*Based on description provided)* |
| “Station Platforms” | 4 - Railways | 1 - Station |
| “Train depot” | 4 - Railways | 0 – Other (train depot) |
| *Named railway line* | 4 - Railways | 0 – Other (mainline route) |
| Technology type |  |  |
| *“Edge-based AI analytics to detect anomalous behaviour”* | 4 – AI Cameras *(based on other descriptions provided)* |  |
| Response activated |  |  |
| *“Police Response”* | 1 – Human Response |  |
| Primary Use |  |  |
| *“All the above”* | *Recoded to reflect all* |  |
| *“Prevent suicide”* | 4 – Prevent Suicide |  |

***Amended responses***

Following discussion and agreement with the team, there were a small number of amendments made to a few sets of responses. Firstly, nine participants indicated that they were not aware of any implemented / planned technology, but answered a small number of questions within the same block (e.g. identifying location). As the survey logic would not allow these questions to be presented if “No” was selected, this indicates that participants subsequently updated their response to “No”. As data were not collected regarding sites without technology/plans for the present work, responses about these nine sites were removed in cleaning and are not included in the analysis.

Furthermore, there were a few instances where participants provided information about a location / technology under another section or reflecting the wrong “stage”. Firstly, one participant had a set of responses moved from “Current Plans” to “Implemented Technology”. This is because, upon reading the free-text responses, it was clear that the technology had already been deployed (rather than something due to happen in the future). Furthermore, four participants entered data about multiple technologies at the same time. Where it was possible to do so, the data were separated and presented as different technologies (and locations where relevant) to ensure accurate counts. Finally, when describing technology deployed at two different locations, one participant indicated several of the responses for location 1 also applied to location 2. The relevant questions were therefore updated to reflect this.

***Missing data***

In one repeated section of the survey (*implemented technology, location 2)* a small number of questions about the location were missing. This affected a single set of responses, and where possible, data were coded based on free-text information provided (i.e. location type and sub-type).
