# Supplementary material for: The use of smart surveillance technologies for suicide prevention in public spaces: a professional stakeholder survey from the United Kingdom
Source: BMC Public Health. 2026 Mar 19;26:1382. doi: 10.1186/s12889-026-26739-0 (PMC13123109; doi:10.1186/s12889-026-26739-0)
Supplement: Supplementary file 3 — Supplementary Material 3. [file 12889_2026_26739_MOESM3_ESM.docx]

**Appendix A**

Full Survey

**Surveillance Technology to Prevent Suicides in Public Places**

**Participant Information Sheet**

**We are very pleased to invite you to take part in a research study that is being conducted by Middlesex University in collaboration with colleagues at the University of Westminster and Samaritans. This information sheet explains why the research is being conducted and what it will involve. Once you have read the information you are welcome to get in touch with the researchers if there is anything that is not clear, or if you would like more information (contact details below). You can take as much time as you like to think about taking part.**

**What is the purpose of the study?**

**The aim of this study is to gather information from key stakeholders across the UK about existing and planned surveillance technologies to prevent suicides in public places.**

**Around 30% of suicides occur in public where prevention would save lives and prevent witness trauma. Recently, the role technology could play in preventing these suicides has come under the spotlight. This includes systems and technologies that may not have been originally or exclusively intended for suicide prevention purposes (e.g. systems to deter or disrupt trespass or antisocial behaviour or to prevent accidents). However, there is little evidence as to whether surveillance technologies can help reduce suicides and suicide attempts in public locations, or if they are ﬁnancially viable. Also, some technologies are controversial (e.g. those that could be used to track people’s movements) so acceptability and ethics are important considerations in this context.**

**This survey is part of a broader study which seeks to ﬁnd out what technology is currently being used, how eﬀective it is in preventing suicides (including understanding any unintended consequences of its use), and how key groups (including stakeholders implementing its use in public places) feel about its impact and acceptability.**

**Please note that our focus in this survey is speciﬁcally on technologies that use sensors, intelligent recognition systems and/or algorithms to process and potentially integrate data (e.g. to then activate an alarm or emergency response). This includes technologies such as automated number plate recognition (ANPR), virtual fencing, infrared sensors and ‘smart cameras’ (excluding standard CCTV technology).**

**Why have I been invited to take part?**

**We are reaching out all local authorities and other relevant stakeholders (e.g. Network Rail, National Highways, the British Parking Association and the British Council of Shopping Centres) and to our contacts within the Safer Public Spaces Network.**

**We are also hoping that people will share a link to our survey within anyone within their networks who might have information about speciﬁc technologies and/or speciﬁc aspects of their current, planned or discontinued use in public locations (e.g. in relation to known or predicted costs). If you know anyone who might be able to help with this survey, please use this** [**link**](https://eu.surveymonkey.com/r/XSWJK6B) **to encourage them to take part: https://eu.surveymonkey.com/r/XSWJK6B**

**Our aim is to reach as many stakeholders as possible, to build a comprehensive picture of the technologies being used in public locations to prevent suicides and identify key lessons on their implementation across diﬀerent types of locations.**

**What will happen if I take part?**

**The study is being conducted online. If you decide you would like to take part, you will be asked to complete an online questionnaire which should take approximately 10 to 20 minutes. You will be asked if you are aware of any ‘intelligent’ surveillance technology currently or previously used in your area/organisation to prevent suicides, trespass, crime/antisocial-behaviour and/or other.**

**Where applicable, brief follow-up questions will focus on perceived risks and beneﬁts, costs and current/discontinued plans. Even if you have no direct experience of implementing or monitoring these technologies, or information about the exact costs involved, it would be really helpful if you could share some information on what is or might be in place at diﬀerent locations, and any thoughts you might have about these technologies.**

**Please let us know if would prefer to discuss this over a brief telephone or video call (rather than via the online survey), and we will be very happy to arrange this at a time that works for you. Our contact details are included at the end of this information sheet.**

**Do I have to take part?**

**No. Participation in this research is entirely voluntary. You do not have to take part if you do not want to. If you decide to take part you may choose not to answer speciﬁc questions, or completely stop or withdraw your participation at any time during the survey, without giving a reason. You can also ask us to delete some or all of your responses within two weeks of taking part in the study (before the data analysis starts). When completing the online survey, you will be given the option to create a unique number. Please contact us at the email address provided below, quoting this number, and we will remove your data. No explanation for your request will be necessary or required.**

**What are the possible advantages and disadvantages to taking part?**

**Our research was funded by the National Institute for Health and Care Research (NIHR) to shape guidance provided to key stakeholders responsible for suicide prevention at high-risk locations across the UK, and support commissioners and policy-makers in making informed decisions about how to invest in suicide prevention, and whether, how, and when this might include surveillance technologies.**

**You will not be asked in this study about any personal experiences of suicide or about speciﬁc incidents you may have encountered as part of your work. However, the survey will include some questions about the beneﬁts and risks of using surveillance technologies to prevent suicides (in general terms). Before deciding if you want to take part in this study, please consider carefully if you are likely to ﬁnd this diﬀicult. For example, it might be better not to take part if you are currently going through a stressful life event or if you are feeling suicidal. If you feel you need help, please click** [**here**](https://www.nhs.uk/mental-health/feelings-symptoms-behaviours/behaviours/help-for-suicidal-thoughts/) **for a list of support organisations.**

**What will happen to the data that I provide by taking part in the survey?**

**Your anonymised responses will be held securely in a ﬁle accessible only to the research team. If you decide to speak to us via telephone or videocall, we will not audio- or videotape our conversation, but will type up some notes which will then be incorporated (also anonymously) in the wider dataset.**

**What will happen to the results of this study?**

**We will produce a summary of the results for the NIHR, who are funding the study, and disseminate key ﬁndings to groups and organisations focusing on suicide prevention (e.g. public health leads, academics and local decision maker, as well as people with lived experience of suicide). The results of this study may also be published in academic journals. Sometimes things that people said might be quoted directly but only if it is not possible to tell from the quotation who said**

**it.**

**You will not be personally identiﬁed or identiﬁable in any report or publication resulting from this study, nor will we name speciﬁc locations or structures in any form of public output.**

**Who has reviewed this study?**

**All proposals for research using human participants are reviewed by an Ethics Committee before they can proceed. The Psychology Department’s Research Ethics Committee at Middlesex University has reviewed and approved this proposal.**

**Contact for further information**

**Thank you for taking the time to read this information. If you have any further questions about the study, please don’t hesitate to contact me via the email, phone or postal address shown below:**

**Professor Lisa Marzano Department of Psychology Middlesex University Town Hall**

**The Burroughs, Hendon London, NW4 4BT**

**Email:** [**L.marzano@mdx.ac.uk**](mailto:L.marzano@mdx.ac.uk)

**You can also download a copy of this participant information sheet by clicking this** [**link**](https://eu.surveymonkey.com/r/XSW56HJ)**.**

* **CONSENT**

Please choose one of the following options:

I have read and understood the information provided and would like to take part in this study.


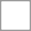
 Yes No

**PART 1. Background information**

Please answer the following questions to help us understand the context of where diﬀerent technologies are being used or might be implemented. Please be reassured that this information will remain anonymous and won’t be used to identify you or your team, or speciﬁc structures or locations.

**(NB. Should you need to close your browser whilst completing the survey, or to pause completion for a little while, you will be able to return to and resume the survey by reopening the link you originally used to access it, from the same device).**

Role

Organisation

Region (Please tick all that apply):

England

Northern Ireland Scotland

Wales

Where in England?

East of England/East Anglia

East Midlands

London

North East

North West

South East (excluding London)

South West

West Midlands

Yorkshire and the Humber

Local Authority

PART 2. Technology currently/previously in use to prevent suicides in public

places

**Intelligent surveillance technologies are advanced computer systems that can gather, analyse and respond to data they collect on an individual’s activities from their surrounding environment (e.g. to then activate an alarm or emergency response). They include automated number plate recognition (ANPR), virtual fencing with infrared sensors or ‘smart cameras’ (excluding standard CCTV).**

- Are you aware of any intelligent surveillance technology currently or previously implemented in your area/organisation to prevent suicides, accidents, trespass, crime or antisocial-behaviour or other?


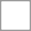
 Yes No

| is technology being used (please tick as many as apply):  to prevent suicide/suicide attempts  to prevent accidental injury or death (e.g. by drowning) to prevent trespass  to prevent crime/anti-social behaviour  Other (please specify) |
| --- |
|  |
| ease tell us a bit more about the location/structure where this technology (or  nologies) are/were being used (Please describe a speciﬁc site/location or location type. u are aware of more than one site implementing such technology, there will be an  on to provide details about further sites later).  re is/was this technology being used?  Bridge  Cliﬀ/Coastal location  Multi-storey structure/high rise building Railways/underground  Park/wooded area/countryside Road  Other (please specify) |
|  |
|  |

Is th

- Pl tech If yo opti

Whe


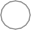

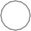

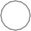

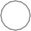

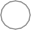

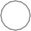

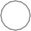


| t type of bridge?  bridge over water bridge over road bridge over railways Other (please specify) |
| --- |
|  |
|  |

Wha


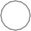

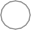

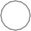

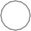


| t type of multi-storey structure/high rise building?  car park  civic or public building commercial building residential building Other (please specify) |
| --- |
|  |
|  |

Wha


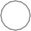

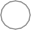

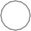

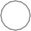

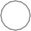


| re on the railways/underground?  Station Crossing Bridge Trackside Tunnel  Other (please specify) |
| --- |
|  |
|  |

Whe


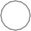

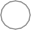

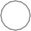

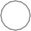

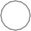

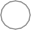


Please specify road-type (e.g. motorway, dual carriage way, etc)

**Please provide the name (if applicable) and postcode or address if known** (please be reassured that we will not share this information in the report, but it is helpful for us to map high-risk locations against nearby structures and risk factors)


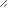


What sort of area is this structure/location in?


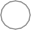
 An urban area/city/town


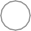
 A rural area/countryside/village
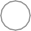
 Other (please specify)

Is access to this site/structure


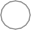
 Public
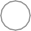
 Private


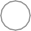
 Communal
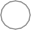
 I don't know


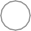
 Other (please specify)

Is this location known locally as a 'high-risk’ or ‘high-frequency’ location for suicide?


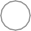
 Yes
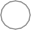
 No


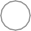
 I don't know

Please use this space to provide any additional information about this location/structure that you think may be useful or signiﬁcant in the context of this study (e.g. size/height/bridge span/number of platforms etc, as applicable):


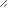


# ABOUT THE TECHNOLOGY

What technology is/was used at this location? *(Please respond to the questions below in relation to a speciﬁc technology. If more than one system is/was used, there will be an option to add further details later)*


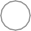
 Automated number plate recognition
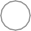
 Bluetooth/Beacon movement tracking


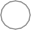
 CCTV activated by movement/proximity (e.g. with infrared/thermal sensors)


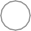
 AI camera with video analytics/‘deep learning’ (e.g. to detect speciﬁc movements, returning persons, anomalous behaviours, etc)


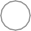
 Drones


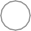
 Virtual fencing/proximity warning systems (e.g. using laser or radar technology, but with no cameras)
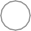
 Other (please specify)

Does this technology activate a human response (e.g. an alert to emergency services and/or CCTV control room), or does it operate as a standalone intervention (e.g. by activating an alarm siren)? (Please tick as many as apply)


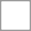
 Human response


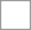
 Standalone intervention
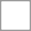
 I don't know

Other (please specify)

Who, if anyone, monitors or monitored its response/outcome/impact? If known/applicable, please provide further details about the process/mechanisms involved:


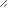


Is/was this technology linked to (please tick all that apply):


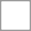
 a two-way audio-system
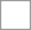
 a one-way audio-system
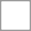
 an audible warning/alarm


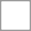
 a visual warning/alarm (e.g. a bright light being activated)
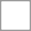
 a call to emergency services or other ﬁrst responders


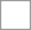
 an alert to the control room (e.g. to monitor the footage)
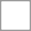
 a text message to the individual/s at the location


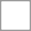
 I don’t know

Other (please specify)

Is/was the technology clearly visible to site visitors?


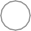
 Yes
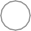
 No


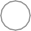
 I don't know

Please provide further details if known/applicable

Is/was this technology advertised through any signage or other?


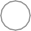
 Yes
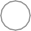
 No


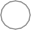
 I don't know

Please provide further details if known/applicable

Is/was this technology primarily used (please tick all that apply):


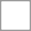
 to prevent accidental injury or death
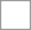
 to prevent trespass


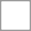
 to prevent crime/anti-social behaviour
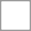
 Other (please specify)

How eﬀective do you perceive this technology to be to prevent suicides at this site?


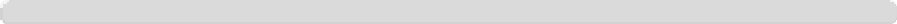

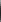

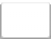

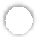

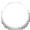


Not at all eﬀective

Very eﬀective

What do you think are/were the 3 main beneﬁts of using this technology to prevent suicides/suicide attempts at this location?

1.

2.

3.

What do you think are/were the 3 main risks or limitations of using this technology to prevent suicides/suicide attempts at this location?

1.

2.

3.

What is or has been the biggest challenge or barrier to using this technology to prevent suicides?


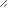


Are you evaluating or planning to evaluate the impact of the technology? If so, how (e.g. by what criteria)? Please let us know if you may be able to share the results of this evaluation


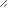


What cost was/is associated with the installation of this technology? Please provide approximate costs, if known:

What ongoing costs are/were associated with the use of this technology? Please provide approximate costs, if known:

How did you ﬁnance this? (e.g. Private funding, part funded by council etc):

If known, when was the technology installed?

Is the technology still in use?


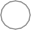
 Yes
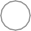
 No


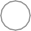
 I don't know

Please provide further information if known (e.g., as applicable, is the technology being used as part of a time-limited trial? Are the plans to extend/expand its use? Why was the technology discontinued? When? Etc. )


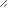


Is there anything else you would like to add in relation to this technology? (E.g. what data is stored, where and for how long for? Who has access to it and who (if anyone) is it shared with?)


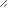


* Are the other technologies at this site you would like to tell us about


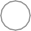

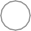
 Yes No

# ABOUT THE TECHNOLOGY (2)

What technology is/was used at this location? *(Please respond to the questions below in relation to a speciﬁc technology. If more than one system is/was used, there will be an option to add further details later)*


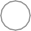
 Automated number plate recognition
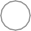
 Bluetooth/Beacon movement tracking


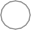
 CCTV activated by movement/proximity (e.g. with infrared/thermal sensors)


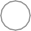
 AI camera with video analytics/‘deep learning’ (e.g. to detect speciﬁc movements, returning persons, anomalous behaviours, etc)


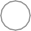
 Drones


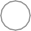
 Virtual fencing/proximity warning systems (e.g. using laser or radar technology, but with no cameras)
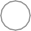
 Other (please specify)

Does this technology activate a human response (e.g. an alert to emergency services and/or CCTV control room), or does it operate as a standalone intervention (e.g. by activating an alarm siren)? (Please tick as many as apply)


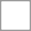
 Human response


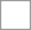
 Standalone intervention
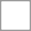
 I don't know

Other (please specify)

Who, if anyone, monitors or monitored its response/outcome/impact? If known/applicable, please provide further details about the process/mechanisms involved:


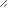


Is/was this technology linked to (please tick all that apply):


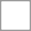
 a two-way audio-system
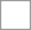
 a one-way audio-system
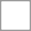
 an audible warning/alarm


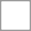
 a visual warning/alarm (e.g. a bright light being activated)
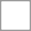
 a call to emergency services or other ﬁrst responders


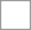
 an alert to the control room (e.g. to monitor the footage)
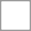
 a text message to the individual/s at the location


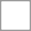
 I don’t know

Other (please specify)

Is/was the technology clearly visible to site visitors?


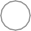
 Yes No

I don't know

Please provide further details if known/applicable

Is/was this technology advertised through any signage or other?

Yes No

I don't know

Please provide further details if known/applicable

Is/was this technology primarily used (please tick all that apply):

to prevent accidental injury or death to prevent trespass

to prevent crime/anti-social behaviour Other (please specify)

How eﬀective do you perceive this technology to be to prevent suicides at this site?

Not at all eﬀective

Very eﬀective

What do you think are/were the 3 main beneﬁts of using this technology to prevent suicides/suicide attempts at this location?

1.

2.

3.

What do you think are/were the 3 main risks or limitations of using this technology to prevent suicides/suicide attempts at this location?

1.

2.

3.

What is or has been the biggest challenge or barrier to using this technology to prevent suicides?

Are you evaluating or planning to evaluate the impact of the technology? If so, how (e.g. by what criteria)? Please let us know if you may be able to share the results of this evaluation

What cost was/is associated with the installation of this technology? Please provide approximate costs, if known:

What ongoing costs are/were associated with the use of this technology? Please provide approximate costs, if known:

How did you ﬁnance this? (e.g. Private funding, part funded by council etc):

If known, when was the technology installed?

Is the technology still in use?

Yes No

I don't know

Please provide further information if known (e.g., as applicable, is the technology being used as part of a time-limited trial? Are the plans to extend/expand its use? Why was the technology discontinued? When? Etc. )

Is there anything else you would like to add in relation to this technology? (E.g. what data is stored, where and for how long for? Who has access to it and who (if anyone) is it shared with?)

* Are the other technologies at this site you would like to tell us about

Yes No

# ABOUT THE TECHNOLOGY (3)

What technology is/was used at this location? *(Please respond to the questions below in relation to a speciﬁc technology. If more than one system is/was used, there will be an option to add further details later)*

Automated number plate recognition Bluetooth/Beacon movement tracking

CCTV activated by movement/proximity (e.g. with infrared/thermal sensors)

AI camera with video analytics/‘deep learning’ (e.g. to detect speciﬁc movements, returning persons, anomalous behaviours, etc)

Drones

Virtual fencing/proximity warning systems (e.g. using laser or radar technology, but with no cameras) Other (please specify)

Does this technology activate a human response (e.g. an alert to emergency services and/or CCTV control room), or does it operate as a standalone intervention (e.g. by activating an alarm siren)? (Please tick as many as apply)

Human response

Standalone intervention I don't know

Other (please specify)

Who, if anyone, monitors or monitored its response/outcome/impact? If known/applicable, please provide further details about the process/mechanisms involved:

Is/was this technology linked to (please tick all that apply):

a two-way audio-system a one-way audio-system an audible warning/alarm

a visual warning/alarm (e.g. a bright light being activated) a call to emergency services or other ﬁrst responders

an alert to the control room (e.g. to monitor the footage) a text message to the individual/s at the location

I don’t know

Other (please specify)

Is/was the technology clearly visible to site visitors?

Yes No

I don't know

Please provide further details if known/applicable

Is/was this technology advertised through any signage or other?

Yes No

I don't know

Please provide further details if known/applicable

Is/was this technology primarily used (please tick all that apply):

to prevent accidental injury or death to prevent trespass

to prevent crime/anti-social behaviour Other (please specify)

How eﬀective do you perceive this technology to be to prevent suicides at this site?

Not at all eﬀective

Very eﬀective

What do you think are/were the three main beneﬁts of using this technology to prevent suicides/suicide attempts at this location?

1.

2.

3.

What do you think are/were the three main risks or limitations of using this technology to prevent suicides/suicide attempts at this location?

1.

2.

3.

What is or has been the biggest challenge or barrier to using this technology to prevent suicides?

Are you evaluating or planning to evaluate the impact of the technology? If so, how (e.g. by what criteria)? Please let us know if you may be able to share the results of this evaluation

What cost was/is associated with the installation of this technology? Please provide approximate costs, if known:

What ongoing costs are/were associated with the use of this technology? Please provide approximate costs, if known:

How did you ﬁnance this? (e.g. Private funding, part funded by council etc):

If known, when was the technology installed?

Is the technology still in use?

Yes No

I don't know

Please provide further information if known (e.g., as applicable, is the technology being used as part of a time-limited trial? Are the plans to extend/expand its use? Why was the technology discontinued? When? Etc. )

Is there anything else you would like to add in relation to this technology? (E.g. what data is stored, where and for how long for? Who has access to it and who (if anyone) is it shared with?)

| t other suicide prevention mitigations, if any, are/were in use at this site? (Please tick hat apply)  Signage Patrols  Staﬀ/gatekeeper training Barriers  I don't know  Other (please specify) |
| --- |
|  |
| e the any other sites/locations implementing technology to prevent suicides you would to tell us about?  Yes No |

Wha all t

* Ar like

# LOCATION 2

**Please provide the name (if applicable) and postcode or address if known** (please be reassured that we will not share this information in the report, but it is helpful for us to map high-risk locations against nearby structures and risk factors)

What sort of area is this structure/location in?

An urban area/city/town

A rural area/countryside/village Other (please specify)

Is access to this site/structure

Public Private

Communal I don't know

Other (please specify)

Is this location known locally as a 'high-risk’ or ‘high-frequency’ location for suicide?

Yes No

I don't know

Please use this space to provide any additional information about this location/structure that you think may be useful or signiﬁcant in the context of this study (e.g. size/height/bridge span/number of platforms etc, as applicable):

## ABOUT THE TECHNOLOGY (location 2)

What technology is/was used at this location? *(Please respond to the questions below in relation to a speciﬁc technology. If more than one system is/was used, there will be an option to add further details later)*

Automated number plate recognition Bluetooth/Beacon movement tracking

CCTV activated by movement/proximity (e.g. with infrared/thermal sensors)

AI camera with video analytics/‘deep learning’ (e.g. to detect speciﬁc movements, returning persons, anomalous behaviours, etc)

Drones

Virtual fencing/proximity warning systems (e.g. using laser or radar technology, but with no cameras) Other (please specify)

Does this technology activate a human response (e.g. an alert to emergency services and/or CCTV control room), or does it operate as a standalone intervention (e.g. by activating an alarm siren)? (Please tick as many as apply)

Human response

Standalone intervention I don't know

Other (please specify)

Who, if anyone, monitors or monitored its response/outcome/impact? If known/applicable, please provide further details about the process/mechanisms involved:

Is/was this technology linked to (please tick all that apply):

a two-way audio-system a one-way audio-system an audible warning/alarm

a visual warning/alarm (e.g. a bright light being activated) a call to emergency services or other ﬁrst responders

an alert to the control room (e.g. to monitor the footage) a text message to the individual/s at the location

I don’t know

Other (please specify)

Is/was the technology clearly visible to site visitors?

Yes No

I don't know

Please provide further details if known/applicable

Is/was this technology advertised through any signage or other?

Yes No

I don't know

Please provide further details if known/applicable

Is/was this technology primarily used (please tick all that apply):

to prevent accidental injury or death to prevent trespass

to prevent crime/anti-social behaviour Other (please specify)

How eﬀective do you perceive this technology to be to prevent suicides at this site?

Not at all eﬀective

Very eﬀective

What do you think are/were the 3 main beneﬁts of using this technology to prevent suicides/suicide attempts at this location?

1.

2.

3.

What do you think are/were the 3 main risks or limitations of using this technology to prevent suicides/suicide attempts at this location?

1.

2.

3.

What is or has been the biggest challenge or barrier to using this technology to prevent suicides?

Are you evaluating or planning to evaluate the impact of the technology? If so, how (e.g. by what criteria)? Please let us know if you may be able to share the results of this evaluation

What cost was/is associated with the installation of this technology? Please provide approximate costs, if known:

What ongoing costs are/were associated with the use of this technology? Please provide approximate costs, if known:

How did you ﬁnance this? (e.g. Private funding, part funded by council etc):

If known, when was the technology installed?

Month/year:

Is the technology still in use?

Yes No

I don't know

Please provide further information if known (e.g., as applicable, is the technology being used as part of a time-limited trial? Are the plans to extend/expand its use? Why was the technology discontinued? When? Etc. )

Is there anything else you would like to add in relation to this technology? (E.g. what data is stored, where and for how long for? Who has access to it and who (if anyone) is it shared with?)

* Are the other technologies at this site you would like to tell us about

Yes No

## ABOUT THE TECHNOLOGY (2, location 2)

What technology is/was used at this location? *(Please respond to the questions below in relation to a speciﬁc technology. If more than one system is/was used, there will be an option to add further details later)*

Automated number plate recognition Bluetooth/Beacon movement tracking

CCTV activated by movement/proximity (e.g. with infrared/thermal sensors)

AI camera with video analytics/‘deep learning’ (e.g. to detect speciﬁc movements, returning persons, anomalous behaviours, etc)

Drones

Virtual fencing/proximity warning systems (e.g. using laser or radar technology, but with no cameras) Other (please specify)

Does this technology activate a human response (e.g. an alert to emergency services and/or CCTV control room), or does it operate as a standalone intervention (e.g. by activating an alarm siren)? (Please tick as many as apply)

Human response

Standalone intervention I don't know

Other (please specify)

Who, if anyone, monitors or monitored its response/outcome/impact? If known/applicable, please provide further details about the process/mechanisms involved:

Is/was this technology linked to (please tick all that apply):

a two-way audio-system a one-way audio-system an audible warning/alarm

a visual warning/alarm (e.g. a bright light being activated) a call to emergency services or other ﬁrst responders

an alert to the control room (e.g. to monitor the footage) a text message to the individual/s at the location

I don’t know

Other (please specify)

Is/was the technology clearly visible to site visitors?

Yes No

I don't know

Please provide further details if known/applicable

Is/was this technology advertised through any signage or other?

Yes No

I don't know

Please provide further details if known/applicable

Is/was this technology primarily used (please tick all that apply):

to prevent accidental injury or death to prevent trespass

to prevent crime/anti-social behaviour Other (please specify)

How eﬀective do you perceive this technology to be to prevent suicides at this site?

Not at all eﬀective

Very eﬀective

What do you think are/were the 3 main beneﬁts of using this technology to prevent suicides/suicide attempts at this location?

1.

2.

3.

What do you think are/were the 3 main risks or limitations of using this technology to prevent suicides/suicide attempts at this location?

1.

2.

3.

What is or has been the biggest challenge or barrier to using this technology to prevent suicides?

Are you evaluating or planning to evaluate the impact of the technology? If so, how (e.g. by what criteria)? Please let us know if you may be able to share the results of this evaluation

What cost was/is associated with the installation of this technology? Please provide approximate costs, if known:

What ongoing costs are/were associated with the use of this technology? Please provide approximate costs, if known:

How did you ﬁnance this? (e.g. Private funding, part funded by council etc):

If known, when was the technology installed?

Month/year:

Is the technology still in use?

Yes No

I don't know

Please provide further information if known (e.g., as applicable, is the technology being used as part of a time-limited trial? Are the plans to extend/expand its use? Why was the technology discontinued? When? Etc. )

Is there anything else you would like to add in relation to this technology? (E.g. what data is stored, where and for how long for? Who has access to it and who (if anyone) is it shared with?)

* Are the other technologies at this site you would like to tell us about

Yes No

## ABOUT THE TECHNOLOGY (3, location 2)

What technology is/was used at this location? *(Please respond to the questions below in relation to a speciﬁc technology. If more than one system is/was used, there will be an option to add further details later)*

Automated number plate recognition Bluetooth/Beacon movement tracking

CCTV activated by movement/proximity (e.g. with infrared/thermal sensors)

AI camera with video analytics/‘deep learning’ (e.g. to detect speciﬁc movements, returning persons, anomalous behaviours, etc)

Drones

Virtual fencing/proximity warning systems (e.g. using laser or radar technology, but with no cameras) Other (please specify)

Does this technology activate a human response (e.g. an alert to emergency services and/or CCTV control room), or does it operate as a standalone intervention (e.g. by activating an alarm siren)? (Please tick as many as apply)

Human response

Standalone intervention I don't know

Other (please specify)

Who, if anyone, monitors or monitored its response/outcome/impact? If known/applicable, please provide further details about the process/mechanisms involved:

Is/was this technology linked to (please tick all that apply):

a two-way audio-system a one-way audio-system an audible warning/alarm

a visual warning/alarm (e.g. a bright light being activated) a call to emergency services or other ﬁrst responders

an alert to the control room (e.g. to monitor the footage) a text message to the individual/s at the location

I don’t know

Other (please specify)

Is/was the technology clearly visible to site visitors?

Yes No

I don't know

Please provide further details if known/applicable

Is/was this technology advertised through any signage or other?

Yes No

I don't know

Please provide further details if known/applicable

Is/was this technology primarily used (please tick all that apply):

to prevent accidental injury or death to prevent trespass

to prevent crime/anti-social behaviour Other (please specify)

How eﬀective do you perceive this technology to be to prevent suicides at this site?

Not at all eﬀective

Very eﬀective

What do you think are/were the 3 main beneﬁts of using this technology to prevent suicides/suicide attempts at this location?

1.

2.

3.

What do you think are/were the 3 main risks or limitations of using this technology to prevent suicides/suicide attempts at this location?

1.

2.

3.

What is or has been the biggest challenge or barrier to using this technology to prevent suicides?

Are you evaluating or planning to evaluate the impact of the technology? If so, how (e.g. by what criteria)? Please let us know if you may be able to share the results of this evaluation

What cost was/is associated with the installation of this technology? Please provide approximate costs, if known:

What ongoing costs are/were associated with the use of this technology? Please provide approximate costs, if known:

How did you ﬁnance this? (e.g. Private funding, part funded by council etc):

If known, when was the technology installed?

Month/year:

Is the technology still in use?

Yes No

I don't know

Please provide further information if known (e.g., as applicable, is the technology being used as part of a time-limited trial? Are the plans to extend/expand its use? Why was the technology discontinued? When? Etc. )

Is there anything else you would like to add in relation to this technology? (E.g. what data is stored, where and for how long for? Who has access to it and who (if anyone) is it shared with?)

| t other suicide prevention mitigations, if any, are/were in use at this site? (Please tick hat apply)  Signage Patrols  Staﬀ/gatekeeper training Barriers  I don't know  Other (please specify) |
| --- |
|  |
|  |

Wha all t

| 3. CURRENT PLANS TO USE SURVEILLANCE TECHNOLOGY TO ENT SUICIDES  e you aware of current plans to use ‘intelligent’ technology in your area/organisation revent suicides, accidents, trespass, crime/antisocial-behaviour or other? (E.g. mated number plate recognition (ANPR), virtual fencing with infrared sensors or  art cameras’, excluding standard CCTV)  Yes No  , please tick as many as apply:  to prevent suicide/suicide attempts  to prevent accidental injury or death (e.g. by drowning) to prevent trespass  to prevent crime/anti-social behaviour  Other (please specify) |
| --- |
|  |
|  |

PART

PREV

* Ar to p auto ‘sm

If so

| ease tell us a bit more about the location/structure where this technology (or  nologies) are/were being used (Please describe a speciﬁc site/location or location type. u are aware of more than one site implementing such technology, there will be an  on to provide details about further sites later).  re is/was this technology being used?  Bridge  Cliﬀ/Coastal location  Multi-storey structure/high rise building Railways/underground  Park/wooded area/countryside Road  Other (please specify) |
| --- |
|  |
|  |

* Pl tech If yo opti

Whe

| t type of bridge?  bridge over water bridge over road bridge over railways Other (please specify) |
| --- |
|  |
|  |

Wha

| t type of multi-storey structure/high rise building?  car park  civic or public building commercial building residential building Other (please specify) |
| --- |
|  |
|  |

Wha

| re on the railways/underground?  Station Crossing Bridge Trackside Tunnel  Other (please specify) |
| --- |
|  |
|  |

Whe

Please specify road-type (e.g. motorway, dual carriage way, etc)

**Please provide the name (if applicable) and postcode or address if known** (please be reassured that we will not share this information in the report, but it is helpful for us to map high-risk locations against nearby structures and risk factors)

What sort of area is this structure/location in?

An urban area/city/town

A rural area/countryside/village Other (please specify)

Is access to this site/structure

Public Private

Communal I don't know

Other (please specify)

Is this location known locally as a 'high-risk’ or ‘high-frequency’ location for suicide?

Yes No

I don't know

Please use this space to provide any additional information about this location/structure that you think may be useful or signiﬁcant in the context of this study (e.g. size/height/bridge span/number of platforms etc, as applicable):

| T THE PLANNED TECHNOLOGY  t technology will be used at this location? *(Please respond to the questions below in tion to a speciﬁc technology. If more than one system is/was used, there will be an on to add further details later)*  Automated number plate recognition Bluetooth/Beacon movement tracking  CCTV activated by movement/proximity (e.g. with infrared/thermal sensors)  AI camera with video analytics/‘deep learning’ (e.g. to detect speciﬁc movements, returning persons, anomalous behaviours, etc)  Drones  Virtual fencing/proximity warning systems (e.g. using laser or radar technology, but with no cameras) Other (please specify) |
| --- |
|  |
| s this technology activate a human response (e.g. an alert to emergency services  /or CCTV control room), or does it operate as a standalone intervention (e.g. by vating an alarm siren)? (Please tick as many as apply)  Human response Standalone intervention I don't know  Other (please specify) |
|  |
| this technology be linked to (please tick all that apply):  a two-way audio-system a one-way audio-system  an audible warning/alarm  a visual warning/alarm (e.g. a bright light being activated) a call to emergency services or other ﬁrst responders  an alert to the control room (e.g. to monitor the footage) a text message to the individual/s at the location  I don’t know  Other (please specify) |
|  |
|  |

# ABOU

Wha *rela opti*

Doe and acti

Will

Will this technology be used primarily (please tick all that apply):

to prevent accidental injury or death to prevent trespass

to prevent crime/anti-social behaviour Other (please specify)

How eﬀective do you perceive this technology to be to prevent suicides at this site?

Not at all eﬀective

Very eﬀective

What do you think are the 3 main beneﬁts of using this technology to prevent suicides/suicide attempts at this location?

1.

2.

3.

What do you think are the 3 main risks or limitations of using this technology to prevent suicides/suicide attempts at this location?

1.

2.

3.

What is the biggest challenge or barrier to using this technology to prevent suicides?

Are you planning to evaluate the impact of the technology? If so, how (e.g. by what criteria)?

What cost will be associated with the installation of this technology? Please provide approximate costs, if known:

What ongoing costs will be associated with the use of this technology? Please provide approximate costs, if known:

How will you ﬁnance this? (e.g. Private funding, part funded by council etc):

How far along is this plan? (e.g. are approvals and funding in place?)

Please use this space to tell us a bit more about these plans (e.g. when is the technology likely to be installed? What data will be gathered? Who will have access to it? Etc.)

* Are there plans to install other technologies at this site?

Yes No

| T THE PLANNED TECHNOLOGY (2)  t technology will be used at this location? *(Please respond to the questions below in tion to a speciﬁc technology. If more than one system is/was used, there will be an on to add further details later)*  Automated number plate recognition Bluetooth/Beacon movement tracking  CCTV activated by movement/proximity (e.g. with infrared/thermal sensors)  AI camera with video analytics/‘deep learning’ (e.g. to detect speciﬁc movements, returning persons, anomalous behaviours, etc)  Drones  Virtual fencing/proximity warning systems (e.g. using laser or radar technology, but with no cameras) Other (please specify) |
| --- |
|  |
| s this technology activate a human response (e.g. an alert to emergency services  /or CCTV control room), or does it operate as a standalone intervention (e.g. by vating an alarm siren)? (Please tick as many as apply)  Human response Standalone intervention I don't know  Other (please specify) |
|  |
| this technology be linked to (please tick all that apply):  a two-way audio-system a one-way audio-system  an audible warning/alarm  a visual warning/alarm (e.g. a bright light being activated) a call to emergency services or other ﬁrst responders  an alert to the control room (e.g. to monitor the footage) a text message to the individual/s at the location  I don’t know  Other (please specify) |
|  |
|  |

# ABOU

Wha *rela opti*

Doe and acti

Will

Will this technology be used primarily (please tick all that apply):

to prevent accidental injury or death to prevent trespass

to prevent crime/anti-social behaviour Other (please specify)

How eﬀective do you perceive this technology to be to prevent suicides at this site?

Not at all eﬀective

Very eﬀective

What do you think are the 3 main beneﬁts of using this technology to prevent suicides/suicide attempts at this location?

1.

2.

3.

What do you think are the 3 main risks or limitations of using this technology to prevent suicides/suicide attempts at this location?

1.

2.

3.

What is the biggest challenge or barrier to using this technology to prevent suicides?

Are you planning to evaluate the impact of the technology? If so, how (e.g. by what criteria)?

What cost will be associated with the installation of this technology? Please provide approximate costs, if known:

What ongoing costs will be associated with the use of this technology? Please provide approximate costs, if known:

How will you ﬁnance this? (e.g. Private funding, part funded by council etc):

How far along is this plan? (e.g. are approvals and funding in place?)

Please use this space to tell us a bit more about these plans (e.g. when is the technology likely to be installed? What data will be gathered? Who will have access to it? Etc.)

* Are there plans to install other technologies at this site?

Yes No

| T THE TECHNOLOGY (3)  t technology will be used at this location? *(Please respond to the questions below in tion to a speciﬁc technology. If more than one system is/was used, there will be an on to add further details later)*  Automated number plate recognition Bluetooth/Beacon movement tracking  CCTV activated by movement/proximity (e.g. with infrared/thermal sensors)  AI camera with video analytics/‘deep learning’ (e.g. to detect speciﬁc movements, returning persons, anomalous behaviours, etc)  Drones  Virtual fencing/proximity warning systems (e.g. using laser or radar technology, but with no cameras) Other (please specify) |
| --- |
|  |
| s this technology activate a human response (e.g. an alert to emergency services  /or CCTV control room), or does it operate as a standalone intervention (e.g. by vating an alarm siren)? (Please tick as many as apply)  Human response Standalone intervention I don't know  Other (please specify) |
|  |
| this technology be linked to (please tick all that apply):  a two-way audio-system a one-way audio-system  an audible warning/alarm  a visual warning/alarm (e.g. a bright light being activated) a call to emergency services or other ﬁrst responders  an alert to the control room (e.g. to monitor the footage) a text message to the individual/s at the location  I don’t know  Other (please specify) |
|  |
|  |

# ABOU

Wha *rela opti*

Doe and acti

Will

Will this technology be used primarily (please tick all that apply):

to prevent accidental injury or death to prevent trespass

to prevent crime/anti-social behaviour Other (please specify)

How eﬀective do you perceive this technology to be to prevent suicides at this site?

Not at all eﬀective

Very eﬀective

What do you think are the 3 main beneﬁts of using this technology to prevent suicides/suicide attempts at this location?

1.

2.

3.

What do you think are the 3 main risks or limitations of using this technology to prevent suicides/suicide attempts at this location?

1.

2.

3.

What is the biggest challenge or barrier to using this technology to prevent suicides?

Are you planning to evaluate the impact of the technology? If so, how (e.g. by what criteria)?

What cost will be associated with the installation of this technology? Please provide approximate costs, if known:

What ongoing costs will be associated with the use of this technology? Please provide approximate costs, if known:

How will you ﬁnance this? (e.g. Private funding, part funded by council etc):

How far along is this plan? (e.g. are approvals and funding in place?)

Please use this space to tell us a bit more about these plans (e.g. when is the technology likely to be installed? What data will be gathered? Who will have access to it? Etc.)

* Are the plans to install technology to prevent suicides at other sites/locations in your area/organisation?

Yes No

## Location 2 of planned technology

**Please provide the name (if applicable) and postcode or address if known** (please be reassured that we will not share this information in the report, but it is helpful for us to map high-risk locations against nearby structures and risk factors)

What sort of area is this structure/location in?

An urban area/city/town

A rural area/countryside/village Other (please specify)

Is access to this site/structure

Public Private

Communal I don't know

Other (please specify)

Is this location known locally as a 'high-risk’ or ‘high-frequency’ location for suicide?

Yes No

I don't know

Please use this space to provide any additional information about this location/structure that you think may be useful or signiﬁcant in the context of this study (e.g. size/height/bridge span/number of platforms etc, as applicable):

| T THE PLANNED TECHNOLOGY (LOCATION 2)  t technology will be used at this location? *(Please respond to the questions below in tion to a speciﬁc technology. If more than one system is/was used, there will be an on to add further details later)*  Automated number plate recognition Bluetooth/Beacon movement tracking  CCTV activated by movement/proximity (e.g. with infrared/thermal sensors)  AI camera with video analytics/‘deep learning’ (e.g. to detect speciﬁc movements, returning persons, anomalous behaviours, etc)  Drones  Virtual fencing/proximity warning systems (e.g. using laser or radar technology, but with no cameras) Other (please specify) |
| --- |
|  |
| s this technology activate a human response (e.g. an alert to emergency services  /or CCTV control room), or does it operate as a standalone intervention (e.g. by vating an alarm siren)? (Please tick as many as apply)  Human response Standalone intervention I don't know  Other (please specify) |
|  |
| this technology be linked to (please tick all that apply):  a two-way audio-system a one-way audio-system  an audible warning/alarm  a visual warning/alarm (e.g. a bright light being activated) a call to emergency services or other ﬁrst responders  an alert to the control room (e.g. to monitor the footage) a text message to the individual/s at the location  I don’t know  Other (please specify) |
|  |
|  |

# ABOU

Wha *rela opti*

Doe and acti

Will

Will this technology be used primarily (please tick all that apply):

to prevent accidental injury or death to prevent trespass

to prevent crime/anti-social behaviour Other (please specify)

How eﬀective do you perceive this technology to be to prevent suicides at this site?

Not at all eﬀective

Very eﬀective

What do you think are the 3 main beneﬁts of using this technology to prevent suicides/suicide attempts at this location?

1.

2.

3.

What do you think are the 3 main risks or limitations of using this technology to prevent suicides/suicide attempts at this location?

1.

2.

3.

What is the biggest challenge or barrier to using this technology to prevent suicides?

Are you planning to evaluate the impact of the technology? If so, how (e.g. by what criteria)?

What cost will be associated with the installation of this technology? Please provide approximate costs, if known:

What ongoing costs will be associated with the use of this technology? Please provide approximate costs, if known:

How will you ﬁnance this? (e.g. Private funding, part funded by council etc):

How far along is this plan? (e.g. are approvals and funding in place?)

Please use this space to tell us a bit more about these plans (e.g. when is the technology likely to be installed? What data will be gathered? Who will have access to it? Etc.)

* Are there plans to install other technologies at this site?

Yes No

| T THE PLANNED TECHNOLOGY (location 2)  t technology will be used at this location? *(Please respond to the questions below in tion to a speciﬁc technology. If more than one system is/was used, there will be an on to add further details later)*  Automated number plate recognition Bluetooth/Beacon movement tracking  CCTV activated by movement/proximity (e.g. with infrared/thermal sensors)  AI camera with video analytics/‘deep learning’ (e.g. to detect speciﬁc movements, returning persons, anomalous behaviours, etc)  Drones  Virtual fencing/proximity warning systems (e.g. using laser or radar technology, but with no cameras) Other (please specify) |
| --- |
|  |
| s this technology activate a human response (e.g. an alert to emergency services  /or CCTV control room), or does it operate as a standalone intervention (e.g. by vating an alarm siren)? (Please tick as many as apply)  Human response Standalone intervention I don't know  Other (please specify) |
|  |
| this technology be linked to (please tick all that apply):  a two-way audio-system a one-way audio-system  an audible warning/alarm  a visual warning/alarm (e.g. a bright light being activated) a call to emergency services or other ﬁrst responders  an alert to the control room (e.g. to monitor the footage) a text message to the individual/s at the location  I don’t know  Other (please specify) |
|  |
|  |

# ABOU

Wha *rela opti*

Doe and acti

Will

Will this technology be used primarily (please tick all that apply):

to prevent accidental injury or death to prevent trespass

to prevent crime/anti-social behaviour Other (please specify)

How eﬀective do you perceive this technology to be to prevent suicides at this site?

Not at all eﬀective

Very eﬀective

What do you think are the 3 main beneﬁts of using this technology to prevent suicides/suicide attempts at this location?

1.

2.

3.

What do you think are the 3 main risks or limitations of using this technology to prevent suicides/suicide attempts at this location?

1.

2.

3.

What is the biggest challenge or barrier to using this technology to prevent suicides?

Are you planning to evaluate the impact of the technology? If so, how (e.g. by what criteria)?

What cost will be associated with the installation of this technology? Please provide approximate costs, if known:

What ongoing costs will be associated with the use of this technology? Please provide approximate costs, if known:

How will you ﬁnance this? (e.g. Private funding, part funded by council etc):

How far along is this plan? (e.g. are approvals and funding in place?)

Please use this space to tell us a bit more about these plans (e.g. when is the technology likely to be installed? What data will be gathered? Who will have access to it? Etc.)

* Are there plans to install other technologies at this site?

Yes No

| T THE PLANNED TECHNOLOGY (location 2)  t technology will be used at this location? *(Please respond to the questions below in tion to a speciﬁc technology. If more than one system is/was used, there will be an on to add further details later)*  Automated number plate recognition Bluetooth/Beacon movement tracking  CCTV activated by movement/proximity (e.g. with infrared/thermal sensors)  AI camera with video analytics/‘deep learning’ (e.g. to detect speciﬁc movements, returning persons, anomalous behaviours, etc)  Drones  Virtual fencing/proximity warning systems (e.g. using laser or radar technology, but with no cameras) Other (please specify) |
| --- |
|  |
| s this technology activate a human response (e.g. an alert to emergency services  /or CCTV control room), or does it operate as a standalone intervention (e.g. by vating an alarm siren)? (Please tick as many as apply)  Human response Standalone intervention I don't know  Other (please specify) |
|  |
| this technology be linked to (please tick all that apply):  a two-way audio-system a one-way audio-system  an audible warning/alarm  a visual warning/alarm (e.g. a bright light being activated) a call to emergency services or other ﬁrst responders  an alert to the control room (e.g. to monitor the footage) a text message to the individual/s at the location  I don’t know  Other (please specify) |
|  |
|  |

# ABOU

Wha *rela opti*

Doe and acti

Will

Will this technology be used primarily (please tick all that apply):

to prevent accidental injury or death to prevent trespass

to prevent crime/anti-social behaviour Other (please specify)

How eﬀective do you perceive this technology to be to prevent suicides at this site?

Not at all eﬀective

Very eﬀective

What do you think are the 3 main beneﬁts of using this technology to prevent suicides/suicide attempts at this location?

1.

2.

3.

What do you think are the 3 main risks or limitations of using this technology to prevent suicides/suicide attempts at this location?

1.

2.

3.

What is the biggest challenge or barrier to using this technology to prevent suicides?

Are you planning to evaluate the impact of the technology? If so, how (e.g. by what criteria)?

What cost will be associated with the installation of this technology? Please provide approximate costs, if known:

What ongoing costs will be associated with the use of this technology? Please provide approximate costs, if known:

How will you ﬁnance this? (e.g. Private funding, part funded by council etc):

How far along is this plan? (e.g. are approvals and funding in place?)

Please use this space to tell us a bit more about these plans (e.g. when is the technology likely to be installed? What data will be gathered? Who will have access to it? Etc.)

PART 4. PAST/DISCONTINUED PLANS TO USE INTELLIGENT SURVEILLANCE

TECHNOLOGY

* Are you aware of past/discontinued plans to use ‘intelligent’ technology in your area/organisation to prevent suicides, accidents, trespass, crime/antisocial-behaviour or other?

(E.g. automated number plate recognition (ANPR), virtual fencing with infrared sensors or ‘smart cameras’, excluding standard CCTV)

Yes No

| this technology going to be used (please tick as many as apply):  to prevent suicide/suicide attempts  to prevent accidental injury or death (e.g. by drowning) to prevent trespass  to prevent crime/anti-social behaviour  Other (please specify) |
| --- |
|  |
| se tell us a bit more about the location/structure where this technology (or nologies) was going to be used (Please describe a speciﬁc site/location or location  . If you are aware of more than one site implementing such technology, there will be ption to provide details about further sites later).  re was this technology going to be used?  Bridge  Cliﬀ/Coastal location  Multi-storey structure/high rise building Railways/underground  Park/wooded area/countryside Road  Other (please specify) |
|  |
|  |

Was

Plea tech type an o

Whe

| t type of bridge?  bridge over water bridge over road bridge over railways Other (please specify) |
| --- |
|  |
|  |

Wha

| t type of multi-storey structure/high rise building?  car park  civic or public building commercial building residential building Other (please specify) |
| --- |
|  |
|  |

Wha

| re on the railways/underground?  Station Crossing Bridge Trackside Tunnel  Other (please specify) |
| --- |
|  |
|  |

Whe

Please specify road-type (e.g. motorway, dual carriage way, etc)

**Please provide the name (if applicable) and postcode or address if known** (please be reassured that we will not share this information in the report, but it is helpful for us to map high-risk locations against nearby structures and risk factors)

What sort of area is this structure/location in?

An urban area/city/town

A rural area/countryside/village Other (please specify)

Is access to this site/structure

Public Private

Communal I don't know

Other (please specify)

Is this location known locally as a 'high-risk’ or ‘high-frequency’ location for suicide?

Yes No

I don't know

Please use this space to provide any additional information about this location/structure that you think may be useful or signiﬁcant in the context of this study (e.g. size/height/bridge span/number of platforms etc, as applicable):

| T THE TECHNOLOGY (PAST/DISCONTINUED PLANS)  t technology was going to be used at this location? *(Please respond to the questions w in relation to a speciﬁc technology. If more than one system is/was used, there will n option to add further details later)*  Automated number plate recognition Bluetooth/Beacon movement tracking  CCTV activated by movement/proximity (e.g. with infrared/thermal sensors)  AI camera with video analytics/‘deep learning’ (e.g. to detect speciﬁc movements, returning persons, anomalous behaviours, etc)  Drones  Virtual fencing/proximity warning systems (e.g. using laser or radar technology, but with no cameras) Other (please specify) |
| --- |
|  |
| s this technology activate a human response (e.g. an alert to emergency services  /or CCTV control room), or does it operate as a standalone intervention (e.g. by vating an alarm siren)? (Please tick as many as apply)  Human response Standalone intervention I don't know  Other (please specify) |
|  |
| this technology going to be linked to (please tick all that apply):  a two-way audio-system a one-way audio-system  an audible warning/alarm  a visual warning/alarm (e.g. a bright light being activated) a call to emergency services or other ﬁrst responders  an alert to the control room (e.g. to monitor the footage) a text message to the individual/s at the location  I don’t know  Other (please specify) |
|  |
|  |

# ABOU

Wha *belo be a*

Doe and acti

Was

Was this technology going to be used primarily (please tick all that apply):

to prevent accidental injury or death to prevent trespass

to prevent crime/anti-social behaviour Other (please specify)

How eﬀective do you perceive this technology to be to prevent suicides at this site?

Not at all eﬀective

Very eﬀective

What do you think are the 3 main beneﬁts of using this technology to prevent suicides/suicide attempts at this location?

1.

2.

3.

What do you think are the 3 main risks or limitations of using this technology to prevent suicides/suicide attempts at this location?

1.

2.

3.

What is the biggest challenge or barrier to using this technology to prevent suicides?

What cost was associated with the installation of this technology? Please provide approximate costs, if known:

How was this going to be ﬁnanced? (e.g. Private funding, part funded by council etc):

When and why were plans to install this technology discontinued?

Please use this space to tell us anything else about these plans or technology (e.g. what data was going to be captured and stored? Who would have had access to it? Etc.)

* Were there plans to install other technologies at this site?

Yes No

| T THE TECHNOLOGY (PAST/DISCONTINUED PLANS 2)  t technology was going to be used at this location? *(Please respond to the questions w in relation to a speciﬁc technology. If more than one system is/was used, there will n option to add further details later)*  Automated number plate recognition Bluetooth/Beacon movement tracking  CCTV activated by movement/proximity (e.g. with infrared/thermal sensors)  AI camera with video analytics/‘deep learning’ (e.g. to detect speciﬁc movements, returning persons, anomalous behaviours, etc)  Drones  Virtual fencing/proximity warning systems (e.g. using laser or radar technology, but with no cameras) Other (please specify) |
| --- |
|  |
| s this technology activate a human response (e.g. an alert to emergency services  /or CCTV control room), or does it operate as a standalone intervention (e.g. by vating an alarm siren)? (Please tick as many as apply)  Human response Standalone intervention I don't know  Other (please specify) |
|  |
| this technology going to be linked to (please tick all that apply):  a two-way audio-system a one-way audio-system  an audible warning/alarm  a visual warning/alarm (e.g. a bright light being activated) a call to emergency services or other ﬁrst responders  an alert to the control room (e.g. to monitor the footage) a text message to the individual/s at the location  I don’t know  Other (please specify) |
|  |
|  |

# ABOU

Wha *belo be a*

Doe and acti

Was

Was this technology going to be used primarily (please tick all that apply):

to prevent accidental injury or death to prevent trespass

to prevent crime/anti-social behaviour Other (please specify)

How eﬀective do you perceive this technology to be to prevent suicides at this site?

Not at all eﬀective

Very eﬀective

What do you think are the 3 main beneﬁts of using this technology to prevent suicides/suicide attempts at this location?

1.

2.

3.

What do you think are the 3 main risks or limitations of using this technology to prevent suicides/suicide attempts at this location?

1.

2.

3.

What is the biggest challenge or barrier to using this technology to prevent suicides?

What cost was associated with the installation of this technology? Please provide approximate costs, if known:

How was this going to be ﬁnanced? (e.g. Private funding, part funded by council etc):

When and why were plans to install this technology discontinued?

Please use this space to tell us anything else about these plans or technology (e.g. what data was going to be captured and stored? Who would have had access to it? Etc.)

* Were there plans to install other technologies at this site?

Yes No

| T THE TECHNOLOGY (PAST/DISCONTINUED PLANS 3)  t technology was going to be used at this location? *(Please respond to the questions w in relation to a speciﬁc technology. If more than one system is/was used, there will n option to add further details later)*  Automated number plate recognition Bluetooth/Beacon movement tracking  CCTV activated by movement/proximity (e.g. with infrared/thermal sensors)  AI camera with video analytics/‘deep learning’ (e.g. to detect speciﬁc movements, returning persons, anomalous behaviours, etc)  Drones  Virtual fencing/proximity warning systems (e.g. using laser or radar technology, but with no cameras) Other (please specify) |
| --- |
|  |
| s this technology activate a human response (e.g. an alert to emergency services  /or CCTV control room), or does it operate as a standalone intervention (e.g. by vating an alarm siren)? (Please tick as many as apply)  Human response Standalone intervention I don't know  Other (please specify) |
|  |
| this technology going to be linked to (please tick all that apply):  a two-way audio-system a one-way audio-system  an audible warning/alarm  a visual warning/alarm (e.g. a bright light being activated) a call to emergency services or other ﬁrst responders  an alert to the control room (e.g. to monitor the footage) a text message to the individual/s at the location  I don’t know  Other (please specify) |
|  |
|  |

# ABOU

Wha *belo be a*

Doe and acti

Was

Was this technology going to be used primarily (please tick all that apply):

to prevent accidental injury or death to prevent trespass

to prevent crime/anti-social behaviour Other (please specify)

How eﬀective do you perceive this technology to be to prevent suicides at this site?

Not at all eﬀective

Very eﬀective

What do you think are the 3 main beneﬁts of using this technology to prevent suicides/suicide attempts at this location?

1.

2.

3.

What do you think are the 3 main risks or limitations of using this technology to prevent suicides/suicide attempts at this location?

1.

2.

3.

What is the biggest challenge or barrier to using this technology to prevent suicides?

What cost was associated with the installation of this technology? Please provide approximate costs, if known:

How was this going to be ﬁnanced? (e.g. Private funding, part funded by council etc):

When and why were plans to install this technology discontinued?

Please use this space to tell us anything else about these plans or technology (e.g. what data was going to be captured and stored? Who would have had access to it? Etc.)

* Were there plans to install technology to prevent suicides at other sites/locations in your area/organisation?

Yes No

## PAST/DISCONTINUED PLANS - location 2

**Please provide the name (if applicable) and postcode or address if known** (please be reassured that we will not share this information in the report, but it is helpful for us to map high-risk locations against nearby structures and risk factors)

What sort of area is this structure/location in?

An urban area/city/town

A rural area/countryside/village Other (please specify)

Is access to this site/structure

Public Private

Communal I don't know

Other (please specify)

Is this location known locally as a 'high-risk’ or ‘high-frequency’ location for suicide?

Yes No

I don't know

Please use this space to provide any additional information about this location/structure that you think may be useful or signiﬁcant in the context of this study (e.g. size/height/bridge span/number of platforms etc, as applicable):

| T THE TECHNOLOGY (DISCONTINUED/PAST PLANS - location 2)  t technology was going to be used at this location? *(Please respond to the questions w in relation to a speciﬁc technology. If more than one system is/was used, there will n option to add further details later)*  Automated number plate recognition Bluetooth/Beacon movement tracking  CCTV activated by movement/proximity (e.g. with infrared/thermal sensors)  AI camera with video analytics/‘deep learning’ (e.g. to detect speciﬁc movements, returning persons, anomalous behaviours, etc)  Drones  Virtual fencing/proximity warning systems (e.g. using laser or radar technology, but with no cameras) Other (please specify) |
| --- |
|  |
| s this technology activate a human response (e.g. an alert to emergency services  /or CCTV control room), or does it operate as a standalone intervention (e.g. by vating an alarm siren)? (Please tick as many as apply)  Human response Standalone intervention I don't know  Other (please specify) |
|  |
| this technology going to be linked to (please tick all that apply):  a two-way audio-system a one-way audio-system  an audible warning/alarm  a visual warning/alarm (e.g. a bright light being activated) a call to emergency services or other ﬁrst responders  an alert to the control room (e.g. to monitor the footage) a text message to the individual/s at the location  I don’t know  Other (please specify) |
|  |
|  |

# ABOU

Wha *belo be a*

Doe and acti

Was

Was this technology going to be used primarily (please tick all that apply):

to prevent accidental injury or death to prevent trespass

to prevent crime/anti-social behaviour Other (please specify)

How eﬀective do you perceive this technology to be to prevent suicides at this site?

Not at all eﬀective

Very eﬀective

What do you think are the 3 main beneﬁts of using this technology to prevent suicides/suicide attempts at this location?

1.

2.

3.

What do you think are the 3 main risks or limitations of using this technology to prevent suicides/suicide attempts at this location?

1.

2.

3.

What is the biggest challenge or barrier to using this technology to prevent suicides?

What cost was associated with the installation of this technology? Please provide approximate costs, if known:

How was this going to be ﬁnanced? (e.g. Private funding, part funded by council etc):

When and why were plans to install this technology discontinued?

Please use this space to tell us anything else about these plans or technology (e.g. what data was going to be captured and stored? Who would have had access to it? Etc.)

* Were there plans to install other technologies at this site?

Yes No

| T THE TECHNOLOGY (PAST/DISCONTINUED PLANS - location 2)  t technology was going to be used at this location? *(Please respond to the questions w in relation to a speciﬁc technology. If more than one system is/was used, there will n option to add further details later)*  Automated number plate recognition Bluetooth/Beacon movement tracking  CCTV activated by movement/proximity (e.g. with infrared/thermal sensors)  AI camera with video analytics/‘deep learning’ (e.g. to detect speciﬁc movements, returning persons, anomalous behaviours, etc)  Drones  Virtual fencing/proximity warning systems (e.g. using laser or radar technology, but with no cameras) Other (please specify) |
| --- |
|  |
| s this technology activate a human response (e.g. an alert to emergency services  /or CCTV control room), or does it operate as a standalone intervention (e.g. by vating an alarm siren)? (Please tick as many as apply)  Human response Standalone intervention I don't know  Other (please specify) |
|  |
| this technology going to be linked to (please tick all that apply):  a two-way audio-system a one-way audio-system  an audible warning/alarm  a visual warning/alarm (e.g. a bright light being activated) a call to emergency services or other ﬁrst responders  an alert to the control room (e.g. to monitor the footage) a text message to the individual/s at the location  I don’t know  Other (please specify) |
|  |
|  |

# ABOU

Wha *belo be a*

Doe and acti

Was

Was this technology going to be used primarily (please tick all that apply):

to prevent accidental injury or death to prevent trespass

to prevent crime/anti-social behaviour Other (please specify)

How eﬀective do you perceive this technology to be to prevent suicides at this site?

Not at all eﬀective

Very eﬀective

What do you think are the 3 main beneﬁts of using this technology to prevent suicides/suicide attempts at this location?

1.

2.

3.

What do you think are the 3 main risks or limitations of using this technology to prevent suicides/suicide attempts at this location?

1.

2.

3.

What is the biggest challenge or barrier to using this technology to prevent suicides?

What cost was associated with the installation of this technology? Please provide approximate costs, if known:

How was this going to be ﬁnanced? (e.g. Private funding, part funded by council etc):

When and why were plans to install this technology discontinued?

Please use this space to tell us anything else about these plans or technology (e.g. what data was going to be captured and stored? Who would have had access to it? Etc.)

## Thank you very much for participating in our study

**Please use this space to record any additional comments (e.g. is there anything else you think we should know about? Are there any additional locations or technologies you would like to tell us about, or any important questions/areas we didn’t ask you about?)**

**Please consider sharing a** [**link**](https://eu.surveymonkey.com/r/XSWJK6B) **to our survey with other people in your networks:**

https://eu.surveymonkey.com/r/XSWJK6B

Would you be interested in being involved in future consultation opportunities relating to this topic (e.g. a follow-up survey to ﬁnd out how diﬀerent technologies and pilots have been progressing in 18 to 24 months, and/or a workshop with other stakeholders to discuss the results of this study)?

Yes No

If you are interested in being involved in future consultation opportunities, please indicate below how we may contact you in due time.

Please note that this does not commit you to anything as you can change your mind at any time. Also, we will not necessarily be able to follow up with everyone who expresses an interest in taking part in further research.

Name

Organisation

I prefer to be contacted via

Telephone

Email

Please be assured that all the information you provided will be stored securely and treated with the strictest conﬁdentiality.

However, if you would like us to permanently delete your responses, please create a unique code (up to 6 digits and/or letters) and contact us quoting this code within the next two weeks (before we start analysing survey responses):

If you would like to withdraw your data NOW please tick the box below:

Yes - I would like to withdraw all my data from the research

No - I would not like to withdraw all my data from the research

Please remove and permanently delete all my survey responses

Yes No

**Should any of our questions have caused you any feelings of distress, or if you would like to ﬁnd out more about services that oﬀer advice and support to people experiencing thoughts of suicide or self-harm, please click** [**here**](https://www.nhs.uk/mental-health/feelings-symptoms-behaviours/behaviours/help-for-suicidal-thoughts/) **for a list of support organisations.**

**If you have any further questions about the research, please don’t hesitate to contact me at the address provided below.**

**Thank you again for your time. Professor Lisa Marzano Department of Psychology Middlesex University**

**Town Hall**

**The Burroughs, Hendon London, NW4 4BT**

**Email:** [**L.marzano@mdx.ac.uk**](mailto:L.marzano@mdx.ac.uk)
