# Supplementary material for: The use of smart surveillance technologies for suicide prevention in public spaces: a professional stakeholder survey from the United Kingdom
Source: BMC Public Health. 2026 Mar 19;26:1382. doi: 10.1186/s12889-026-26739-0 (PMC13123109; doi:10.1186/s12889-026-26739-0)
Supplement: Supplementary file 4 — Supplementary Material 4. [file 12889_2026_26739_MOESM4_ESM.docx]

**Appendix B**

**Coding Frame**

^1^ANPR = automatic number place recognition

^2^VF = virtual fencing

^3^BB = Bluetooth beacon

^4^AI cam = Artificial intelligence camera

^5^MA CCTV = motion activated CCTV

| **What do you think are/were the 3 main *benefits* of using this technology to prevent suicides/suicide attempts at this location?** | | | | | | | | | |
| --- | --- | --- | --- | --- | --- | --- | --- | --- | --- |
| **Category** | **Quotes** | **Location** | | | | | | | |
|  |  | **Tech** | | | | | | | |
| **Surveillance technologies provide data to initiate an accurate and appropriate response** | | | | | | | | | |
| Early identification and a fast response | Enable a quick security response before the person can put themselves in danger (P6 – virtual fence)  It alerts the emergency services immediately. (P12 – ANPR)  Opportunities to interveve [sic] early (P13 – MA CCTV)  Early alert to the CSC's (P25 – AI cameras)  Standardised process for alerting, Signalers( to implement live instruction/cautions to drivers in the vicinity [sic], BTP, Emergency Services and Control. (P25 – AI cameras)  Allows a quicker response (P28 – MA CCTV)  Early notification of persons in distress enabling rapid response and intervention (P42 – other: Edge-based AI analytics to detect anomalous behaviour)  Quick response (P45 – other: surveillance system)  Additional time to respond (P50 – MA CCTV)  Police aware of incidents more quickly (P53 – ANPR)  Earlier intervention chance to help them (P71, other)  Human intervention (P77, AI camera)  Control rooms can alert trains and station staff to vulnerable people (P90 – MA CCTV)  Provides a rapid licensed attendance in case of confirmed activity (P83- MA CCTV; AI camera)  Early indicator to Control Room Staff (P106 – AI camera)  Early intervention enabler (P106 – AI camera)  Response times (P107 – drones)  Early notification of the stopped vehicle (P122 – other: Infra-red devices able to identify vehicles stopped when the traffic is still flowing)  Opportunity to dispatch Traffic Officers to the scene (P122 – other: Infra-red devices able to identify vehicles stopped when the traffic is still flowing)  Early awareness of vulnerable person (P123 – AI camera) | **Bridge** | **Rail** | **Coast** | **High rise** | **Road** | **Park** | **Other** | **Total** |
|  |  | 7 | 4 | 1 | 3 | 3 | 0 | 1 | **20** |
|  |  | **ANPR^1^** | **VF^2^** | **BB^3^** | **AI cam^4^** | **MA CCTV^5^** | **Drone** | **Other** | **Total** |
|  |  | 2 | 1 |  | 7 | 5 | 1 | 5 | **21** |
| Increase intervention opportunities | to prevent through direct communication (P8 – MA CCTV)  increase opportunity for human intervention (P51 – other: a few different bits)  Potential to intervene with a vulnerable person (P123 – bluetooth beacon)  Allowing intervention from staff at location (P126 – virtual fence)  increased opportunity to intervene (P130 – MA CCTV) | 3 | 1 | 0 | 1 | 0 | 0 | 0 | **5** |
|  |  | 0 | 1 | 1 | 0 | 2 | 0 | 1 | **5** |
| More accurate / appropriate response/ intervention | If someone did jump, cameras could automatically locate and 'lock on' to the person in the water, enabling a better SAR response when every second is critical. (P42 – other: Edge-based AI analytics to detect anomalous behaviour)  Able to call the most appropriate services (P107, drone)  Ability to deploy responders more accurately (P116 – MA CCTV)  targeted location for possible intervention (P123 – AI camera) | 2 | 0 | 1 | 0 | 1 | 0 | 0 | **4** |
|  |  | 0 | 0 | 0 | 1 | 1 | 1 | 1 | **4** |
| Reducing suicide | this does result in a reduction in suicide (P45 – other: This is a surveillance system manned by employees)  reduction in suicides (P71 –other)  reduction in deaths (P130 – MA CCTV)  reduction in attemptss (P130 – MA CCTV) | 3 | 0 | 0 | 0 | 0 | 0 | 1 | **4** |
|  |  | 0 | 0 | 0 | 0 | 2 | 0 | 2 | **4** |
| Reduce impact on others | Decrease disruption to local economy. (P13- MA CCTV)  Ability to set signs and signals to warn approaching traffic of the hazzard (P122 – other: Infra-red devices able to identify vehicles stopped when the traffic is still flowing) | 1 | 0 | 0 | 0 | 1 | 0 | 0 | **2** |
|  |  | 0 | 0 | 0 | 0 | 1 | 0 | 1 | **2** |
| Notification/ alert someone is where they shouldn’t be | Awareness that someone had passed through the gate from the public accessible side of the platform (P5 – MA CCTV)  to alert stakeholders - train cautioning (P8 – MA CCTV)  to alert responders (P8 – MA CCTV)  reported activity (P8 – MA CCTV)  can spot persons in quiet areas (P14 –MA CCTV)  Helps identify persons in high risk areas (P28 – MA CCTV)  Alerts the control room team (P28 –MA CCTV)  Monitoring centre can see any issues (P33 – ANPR for access charging,; MA CCTV)  Awareness of individuals of the footpaths (P50 – MA CCTV)  Alarms straight into the control room (P50- MA CCTV)  better situational awareness (P51 – other: a few different bits)  Incidents can be filtered by control room, situations where inadvertent breaches of cordon can be dismissed (P53 – MA CCTV)  Will alert Police Control Room to persons in areas they should not be (P70, MA CCTV)  proven technology to identify line crossing by vulnerable people (P123 – radar) | **Bridge** | **Rail** | **Coast** | **High rise** | **Road** | **Park** | **Other** | **Total** |
|  |  | 5 | 4 | 0 | 3 | 0 | 0 | 2 | **14** |
|  |  | **ANPR** | **VF** | **BB** | **AI cam** | **MA CCTV** | **Drone** | **Other** | **Total** |
|  |  | 0 | 0 | 0 | 0 | 12 | 0 | 2 | **14** |
| Identify & monitor at-risk people | Real time observation of persons at risk (P9 – other: technology to digitally observe electronic devices)  Detection of historic presenters when they return (P9 – other: technology to digitally observe electronic devices)  Identify repeat presentations (P13 – MA CCTV)  can be used to identify vulnerable persons (P14 – MA CCTV)  track and trace vulnerable persons movements if onsite (P14 –MA CCTV)  linked to emergency services (P115 –ANPR) | 1 | 0 | 0 | 2 | 0 | 0 | 3 | **6** |
|  |  | 1 | 0 | 0 | 0 | 3 | 0 | 2 | **6** |
| Learning | AI to determine risk factors for suicide and crime (P9 – other: technology to digitally observe electronic devices)  Intelligence to improve sites (Suicide Safer ) Building in measures early with any new planning decisions to prevent issues at new sites.(p13 – MA CCTV)  We have learned a lot of the pitfalls from the trials (P40 – AI cameras)  We have evidence for future trials (P40 – AI cameras)  Whilst the trials have been inconclusive in effectiveness we can use this to improve future projects. (P40 – AI cameras)  Key learning from data (P45 –other: This is a surveillance system manned by employees)  Reflective learning on what action can be taken to prevent incidents (P107, drone)  New knowledge of movements on the cliffs (P116 – MA CCTV) | 2 | 3 | 1 | 0 | 1 | 0 | 1 | **8** |
|  |  | 0 | 0 | 0 | 3 | 2 | 1 | 2 | **8** |
| Provides data, including trends & patterns | better understanding of patterns at High risk locations (P51 – other: a few different bits)  the number using that area (P71 – other: a mixture)  Provides situational awareness of the premises and human interactions with it (P83 –MA CCTV; AI camera)  It lets us see circumstances surrounding an event. (P90 – MA CCTV)  It will help us understand the pre-jump behaviour of individuals (P116 – Bluetooth beacon)  It will identify any common behaviours that can be used as markers for very high jumping risk. (P116 – Bluetooth beacon)  It will increase surveillance to help us understand how all people move across the cliffs (P116 – Bluetooth beacon) | 1 | 1 | 3 | 1 | 0 | 0 | 1 | **7** |
|  |  | 0 | 0 | 3 | 1 | 2 | 0 | 2 | **8** |
| Adding/linking data/systems/ organisations | additional layer of surveillance (P14 –MA CCTV)  Possibility of linkage to reports of missing individuals / stress situations (P53 – ANPR)  Integration with ANPR surveillance (P53 – MA CCTV)  Any vehicles which had information markers on them pertaining to a concern for welfare of the driver, which had been placed on the vehicle anywhere in the United Kingdom would alert the Port Police if this vehicle entered the Port. (P70, ANPR)  linked to emergency services (P115 –ANPR)  Potential to share data with other relevant public bodies (P123 – Bluetooth beacon)  Hopefully alert hospital staff about people in areas they ought not to access (P126 –bluetooth beacon) | 3 | 0 | 0 | 2 | 0 | 0 | 2 | **7** |
|  |  | 3 | 0 | 2 | 0 | 2 | 0 | 0 | **7** |
| **Technology should meet the needs of the location** | | | | | | | | | |
| Discrete | It is discreet - it prevents knowledge of the site being a high risk location. (P12 – ANPR)  It is hidden unless it is triggered (P90 – online interceptive tool) | **Bridge** | **Rail** | **Coast** | **High rise** | **Road** | **Park** | **Other** | **Total** |
|  |  | 0 | 1 | 1 | 0 | 0 | 0 | 0 | **2** |
|  |  | **ANPR** | **VF** | **BB** | **AI cam** | **MA CCTV** | **Drone** | **Other** | **Total** |
|  |  | 1 | 0 | 0 | 0 | 0 | 0 | 1 | **2** |
| Accessible | The technology is accessible for a remote coastal site with limited infrastructure / reception. (P12 – ANPR) | 0 | 0 | 1 | 0 | 0 | 0 | 0 | **1** |
|  |  | 1 | 0 | 0 | 0 | 0 | 0 | 0 | **1** |
| Easy to use | easy to use (P14 –MA CCTV) | 0 | 0 | 0 | 1 | 0 | 0 | 0 | **1** |
|  |  | 0 | 0 | 0 | 0 | 1 | 0 | 0 | **1** |
| Improves upon human involvement | less reliant on people (P14 –MA CCTV)  With over 8000 cameras , live detection is unlikely. (P25 – AI cameras)  Drones have been finding suicidal people after they have hidden from foot patrols. (P40 – drones)  Drones can declare the lines free quicker than a foot patrol and enable trains to run again. (P40 – drones)  Drones have thermal imaging capability and a long range. (P40 – drones)  Reducing CCTV operator burden through automated, 24/7 monitoring (P42 –other: Edge-based AI analytics to detect anomalous behaviour)  24/7 patrolling (P115 – ANPR)  Extra pairs of eyes as responders are often already engaged in a rescue (P116 – MA CCTV)  Automated to a certain extent (P123 – ANPR) | 1 | 4 | 1 | 1 | 1 | 0 | 1 | **9** |
|  |  | 2 | 0 | 0 | 1 | 2 | 3 | 1 | **9** |
| Multi-purpose | Crowd Management (P39 –MA CCTV)  Pedestrianise Safety (P39 –MA CCTV)  Multi-functional including crime prevention (P123 – ANPR) | 0 | 0 | 0 | 0 | 1 | 2 | 0 | **3** |
|  |  | 1 | 0 | 0 | 0 | 2 | 0 | 0 | **3** |
| Deterrent | very loud to deter access (P8 – MA CCTV)  Audio deterrent (P77, AI camera)  Signage may deter attempts to enter the area (P83 –MA CCTV; AI camera)  Visual deterrent (P126 –virtual fence) | 0 | 2 | 0 | 2 | 0 | 0 | 0 | **4** |
|  |  |  | 1 |  | 2 | 2 |  |  | **5** |
| Prevent access | prevented access (P8 – MA CCTV)  Preventing access to dangerous area (P126 –virtual fence)  The use of technology sounds like a brilliant, innovative way to progress reducing access to means (P26 – additional comments) | 0 | 1 | 0 | 2 | 0 | 0 | 0 | **2** |
|  |  | 0 | 1 | 0 | 0 | 1 | 0 | 0 | **2** |

| **What do you think are/were the 3 main *risks or limitations* of using this technology to prevent suicides/suicide attempts at this location?**  **What is or has been the biggest challenge or barrier to using this technology to prevent suicides?**  **Please use this space to record any additional comments (e.g. is there anything else you think we should know about? Are there any additional locations or technologies you would like to tell us about, or any important questions/areas we didn’t ask you about?)** | | | | | | | | | |
| --- | --- | --- | --- | --- | --- | --- | --- | --- | --- |
| **Category** | **Quotes** | **Location** | | | | | | | |
|  |  | **Tech** | | | | | | | |
| **Challenges of Surveillance Technologies Requiring a Human Response to Prevent Suicide** | | | | | | | | | |
| Still requires human response which can be too slow/ have errors | Reaction not instant (P5 – MA CCTV)  Person receiving notification has to then contact all relevant teams. (P5 – MA CCTV)  Still requires a human intervention, so is open to human error. (P6 –Virtual fence)  lack of response by monitoring people (P8 – MA CCTV)  poor monitoring of system (P8 – MA CCTV)  poor management of statistics of effectiveness (P8 – MA CCTV)  The Police will not always search the ANPR database immediately when searching for a vulnerable person. (P12 – ANPR)  can be missed by monitoring team (P14 –MA CCTV)  Still requires human intervention (P28 –MA CCTV)  Only effective if picked up be [sic] controller (P28 –MA CCTV)  Getting an effective and timely response plan in place. (P40 – AI camera)  the tech is only as good as the people monitoring it (P51 – other: a few different bits)  Camera must be viewed live by an operator (P70 – MA CCTV)  The rapidity of any response would be insufficient to address a determined planned attempt (P83 –MA CCTV; AI camera)  it relies on observation by a third party (P90 – MA CCTV)  It relies on a human assessing the situation (P90 – MA CCTV)  Ensuring effective monitoring and response timeframes to safety (P107 – drones)  By the time a unit has been dispatched to the scene it is often too late (P122 – other: Infra-red devices able to identify vehicles stopped when the traffic is still flowing)  The suicide events on the [REDACTED] Bridge tend to be swift.(P122 – other; Infra-red devices able to identify vehicles stopped when the traffic is still flowing)  reliance on human response (P130 – MA CCTV)  Early stages but think its about who has capacity to monitor these 24hours. Needs to be some kind of resource that can deployed. (P13 – MA CCTV)  human error, (P14 –MA CCTV)  Not actively monitored (P33 – MA CCTv)  Getting a proper, timely response plan in place for when activations occur. Smart Cameras can go in but who responds when they send an alert? This is harder to organise than cameras (P40 -AI camera)  Response times. (P40 -drones)  responsibility of continuous monitoring (P130 -MA CCTV) | **Bridge** | **Rail** | **Coast** | **High rise** | **Road** | **Park** | **Other** | **Total** |
|  |  | 4 | 10 | 1 | 6 | 3 | 0 | 2 | **26** |
|  |  | **ANPR** | **VF** | **BB** | **AI cam** | **MA CCTV** | **Drone** | **Other** | **Total** |
|  |  | 1 | 1 | 0 | 3 | 17 | 2 | 3 | **27** |
| Impact on staff from having to monitor/witness suicides | Those exposed to been involved, E.g people in control rooms or staff sent to the location (P13 – MA CCTV)  Staff wellbeing (when incidents occur) (P45 – other: This is a surveillance system manned by employees) | 2 |  |  |  |  |  |  | **2** |
|  |  |  |  |  |  | 1 |  | 1 | **2** |
| Staff resistance to new tech | Staff resistance to use of new systems and equipment. (P40 – AI camera) |  | 1 |  |  |  |  |  | **1** |
|  |  |  |  |  | 1 |  |  |  | **1** |
| Staff training required | Getting enough people trained. (P40 – drones) |  | 1 |  |  |  |  |  | **1** |
|  |  |  |  |  |  |  | 1 |  | **1** |
| **Ethical and Legal Concerns** | | | | | | | | | |
| Displacement of attempts to other locations | displacing the attempt (P8 – MA CCTV)  Could potentially 'displace' suicide attempts to another location if technology becomes widely known (P42 – other: Edge-based AI analytics to detect anomalous behaviour; other: Advanced object detection and recognition for a range of potential threats e.g. weapons. Any potential safety or security use case identified by the end-user; AI cameras)  People may go elsewhere to end their life (P126 –virtual fence) | **Bridge** | **Rail** | **Coast** | **High rise** | **Road** | **Park** | **Other** | **Total** |
|  |  | 1 | 1 |  | 1 |  |  | 1 | **4** |
|  |  | **ANPR** | **VF** | **BB** | **AI cam** | **MA CCTV** | **Drone** | **Other** | **Total** |
|  |  |  | 1 |  | 1 | 1 |  | 1 | **4** |
| May draw more attention to the site as a suicide location | increase notoriety of location as a suicide opportunity (P130 – MA CCTV) | 1 |  |  |  |  |  |  | **1** |
|  |  |  |  |  |  | 1 |  |  | **1** |
| Impact on families finding out their loved ones death was monitored | The covert nature of the surveillance and the reputational risk if this is discovered, which would then also highlight to bereaved relatives that their loved ones deaths were being monitored. .(P116 – Bluetooth beacon) |  |  | 1 |  |  |  |  | **1** |
|  |  |  |  | 1 |  |  |  |  | **1** |
| Legality / data protection issues | Peoples misunderstanding of the GDPR in relation to the legal use of the technology (P9 – other: technology to digitally observe electronic devices)  The pilot would rely on covert use of technology which may not be legally feasible (P116 – Bluetooth beacon)  The pilot would have to observe real deaths in real time without intervention. this is ethically difficult. (P116 – Bluetooth beacon)  Although I am of course keen to prevent suicides, I am very concerned about the use of these technologies generally, as well as for this purpose. I think this raises many concerns about human rights, freedoms, and much more. Generally, there is far too much use already of systems such as this, decreasing much needed and very beneficial human contact, e.g. in supermarkets. I would be very concerned about using similar for suicide prevention. I think this is a very complex area that would require a huge amount of research and very serious consideration. Otherwise, like the use of these systems elsewhere, it could do more harm than good. Thank you (P101 – additional comments) |  |  | 2 |  |  |  | 1 | **3** |
|  |  |  |  | 2 |  |  |  | 1 | **3** |
| **Surveillance Technologies Alone are Not Enough to Prevent Suicides** | | | | | | | | | |
| Humans > tech when it comes to suicide prevention / possibility for over reliance on tech | It is not monitored by a person (P1 –ANPR)  humans are always better than technology when it comes to preventing suicides (P51 – other: a few different bits)  becoming too reliant on tech (P14 –MA CCTV)  Overall when using systems such as AI to support in the prevention of suicide we still have to understand that AI is not perfect. There is also the issue of bias when creating a programme to single out groups of people in public spaces in this case those who are experiencing suicidal thoughts. How does the programme decide who does or does not fit in that category and what steps are then taken to prevent the immediate risk. We know that preventing immediate risk of suicide is important but that currently relies on an overstretched system of emergency services. Perhaps time and energy would be better focused on longer term prevention programmes, focusing on high risk individuals before they get to the place where they are at immediate risk of suicide. (P79 – additional comments) | **Bridge** | **Rail** | **Coast** | **High rise** | **Road** | **Park** | **Other** | **Total** |
|  |  | 1 |  |  | 2 |  |  |  | **3** |
|  |  | **ANPR** | **VF** | **BB** | **AI cam** | **MA CCTV** | **Drone** | **Other** | **Total** |
|  |  | 1 |  |  |  | 1 |  | 1 | **3** |
| Not designed with suicide in mind / not its primary purpose | It is not used to prevent suicides (P79 - MA CCTV)  System coverage is not established in order to be preventative of suicide, but to detect intrusion. (P83 –MA CCTV; AI camera)  It is not designed with suicide prevention in mind (P122 – other: Infra-red devices able to identify vehicles stopped when the traffic is still flowing)  It is for a different purpose (P1 –ANPR)  Very effective system in deterring trespass, difficult to ascertain who was trespass, thief or suicidal. So hard to build statistics for suicide prevention (P8 – MA CCTV)  Drones are being used by the MCGA and Border force for migrant activity. However I do not have any data to understand if it is used in Suicide prevention. (P12 – drones)  not used to prevent suicide (P36 –ANPR)  It is not the principle purpose of the surveillance, which is to detect intrusion. There is a certain applicable benefit that coincides with suicide risk mitigation, but the principle purpose has to be prioritised as it is a paid for service that has been customer specified (P83 –MA CCTV; AI Camera)  It isn't the primary role of stopped vehicle technology to help detect or prevent suicide. (P122 – other: Infra-red devices able to identify vehicles stopped when the traffic is still flowing) |  | 1 | 1 | 5 | 2 |  |  | **9** |
|  |  | 2 |  |  | 2 | 4 | 1 | 2 | **11** |
| Retrospective capabilities, not preventative | Doesn't physically prevent anything happening (P53 – ANPR)  Again, the technology isn't guaranteed to prevent incidents of self-harm (P53 – MA CCTV)  It is exclusively a retrospective rather than a proactive measure (P83 –MA CCTV; AI camera)  It's not a preventative measure for suicide risk for the individual, (P90 – MA CCTV)  Failing to proactively identify a suicide risk using Smart technology for people to contact in a crisis (P107 – drones)  Limited support contacts for people at a high risk of suicide. (P107 – drones)  Prevention is better proactively however this technology leads to a chain of provisions that could be too late when a person is intent on harming themselves. (P107 – drones) | 2 | 1 |  | 1 | 3 |  |  | **7** |
|  |  | 1 |  |  | 1 | 3 | 3 |  | **8** |
| More info is needed | They are not connected to anything/ anyone (P68 – other: LoRaWAN People Counter)  Only where a vehicle is involved, infrequent in [REDACTED] (P123 – ANPR)  Knowledge of the individual's vehicle (P123 – ANPR)  No help if there is no record of the vulnerable person on the database (P123 – Bluetooth beacon)  One system alone will not resolve the issue, but other measures are being considered to make the site safer (P126 –Bluetooth beacon)  vehicle registrations not shared (P14 –ANPR)  Requires a marker being placed on a vehicle to raise a concern for the driver/occupant. (P70 – ANPR) | 2 |  |  | 2 | 2 |  | 1 | **7** |
|  |  | 4 |  | 2 |  |  |  | 1 | **7** |
| People can get round tech / It may not be accessed | It will only flag vehicles where the person is being looked for. (P12 – ANPR)  People who have plans are able to jump can do so at ease if there is no time in-between reaching the location and jumping, Barriers remain low (P13 – MA CCTV)  person could park and leave vehicle to relocate (P14 –ANPR)  no person specific (P14 –ANPR)  vulnerable persons could potentially avoid being tracked (P14 –MA CCTV)  Some suicides are not preventable. Instant actions can not be managed. (P25 – AI cameras)  Individuals becoming aware of the sensors, trying to bypass (P50 – MA CCTV)  People need to be on the WiFi and accessing content on the block list (P90 – online interceptive tool)  At major stations, mobile data coverage is usually strong so the WiFi may not be used (P90 – online interceptive tool)  The block list may be incomplete so people can slip through the net. (P90 – online interceptive tool)  Determined people may still scale the fence or find ways around (P126 –virtual fence)  Capturing the right people through our WiFi and getting them help (P90 – online interceptive tool) | 2 | 5 | 1 | 4 |  |  |  | **12** |
|  |  | 3 | 1 |  | 1 | 3 |  | 4 | **12** |
| **Practical Barriers to Installing Surveillance Technologies** | | | | | | | | | |
| Costs | Its funding and maintenance is reliant on [REDACTED] police. (P12 – ANPR)  Cameras can be bought but ongoing monitoring funds are harder to obtain (P40- AI camera)  Costs of the equipment and training are high (P40 – drones)  Costing (P71 – other: a mixture)  Costs (P130 – MA CCTV)  Cost (P28 –MA CCTV)  Cost (P39 – MA CCTV)  Unfortunately the technology was withdrawn before it became operational due to the costs of producing the beacons (P119 –Bluetooth beacon) | **Bridge** | **Rail** | **Coast** | **High rise** | **Road** | **Park** | **Other** | **Total** |
|  |  | 1 | 2 | 1 | 2 |  | 1 | 1 | **8** |
|  |  | **ANPR** | **VF** | **BB** | **AI cam** | **MA CCTV** | **Drone** | **Other** | **Total** |
|  |  | 1 |  | 1 | 1 | 3 | 1 | 1 | **8** |
| Tech issues | not loud enough (P8 – MA CCTV)  could fail (P14 – MA CCTV)  Software or power outage. (P25 – AI cameras)  Speed of deployment can be a limitation (P40 – drones)  Network - alarm not transmitted to contol room due to poor network (P59 – other: Video Analytics, AI object classification, Radar)  connecting it to our systems/servers (P51 – other: a few different bits)  The equipment is not sufficiently robust for deployment on a bridge over water (P123 -radar)  Installing the correct system with features that allow this to work smoothly (P126 - Bluetooth beacon) | 2 | 4 |  | 2 |  |  |  | **8** |
|  |  |  |  | 1 | 1 | 2 | 1 | 3 | **8** |
| Visibility & coverage | Visibility in the dark (P5 – MA CCTV)  not all areas covered (P14 –MA CCTV)  Specification may need to be adjusted due to seasons. ( Shadows, foliage and reflections may trigger false alarms) (P25 – AI Cameras)  Limited visual scope (P33 – ANPR for access charging, CCTV for security; MA CCTV)  May not work as well in dark conditions unless there is enough of a thermal imagery dataset to train the AI Early notification of persons in distress enabling rapid response and intervention (P42 – other: Edge-based AI analytics to detect anomalous behaviour)  Limited range of two way audio (P53 – ANPR)  Lack of coverage - impossible to cover all the tracks. (P59 – other: Video Analytics, AI object classification, Radar)  ANPR cameras are only present at the entry points to the Port, other public areas in the vicinity do not have this technology present. (P70 – ANPR)  someone has to be watching that exact spot they intend to use (P71- other: a mixture)  Does not cover all locations across the District (P115 – ANPR)  Will it truly have the range/ ability to see as much as thought? (P116 – MA CCTV)  not 100% coverage (MA CCTV)  probably not viewing the most dangerous areas (P33 –ANPR for access charging, CCTV for security, MA CCTV)  Not all areas where persons in crisis can access dangerous locations are covered by motion detecting cameras (P70- MA CCTV)  That it simply does not show enough detail (P116 – MA CCTV) | 2 | 3 | 2 | 2 |  |  | 7 | **16** |
|  |  | 3 |  |  | 2 | 8 |  | 3 | **16** |
| Risk of damage | Vandalism (P8 – MA CCTV)  Damage to the sensors, vandalism (P50 – MA CCTV)  Exposure of technology to the elements (P116 – MA CCTV)  It works well but has been subject to minor vandalism. (P12 – ANPR) | 1 | 1 | 2 |  |  |  |  | **4** |
|  |  | 1 |  |  |  | 3 |  |  | **4** |
| Public perception | Public perceptions of being monitiored [sic] (P116- MA CCTV) |  |  | 1 |  |  |  |  | **1** |
|  |  |  |  |  |  |  | 1 |  | **1** |
| Visible (unappealing) | visible could move them to a different location (P71 – a mixture)  Unattractive measure, though this may be enhanced using something more aesthetically pleasing over time (P126 –virtual fence) |  |  |  | 1 |  |  | 1 | **2** |
|  |  |  | 1 |  |  |  |  | 1 | **2** |
| False alarms | Multiple false activations leading to apathy in response. (P40 – AI cameras)  Potential for high false alarm rate unless algorithm is correctly refined. Early notification of persons in distress enabling rapid response and intervention (P42 – other: Edge-based AI analytics to detect anomalous behaviour)  On the open footpath alarms are triggered all day by public (P50 – MA CCTV)  the tech is not infallible from what i have heard eg: thermal cameras can pick up all sorts of things that we do not want them to (P51 – other: a few different bits)  False alarm - alarm generated by events determined but prove to be false. (P59 – other: Video Analytics, AI object classification, Radar)  False positives (e.g. animal detected) cause fatigue with notifications (P90 - MA CCTV)  False alarms (P106 – AI camera)  Many false positives (P123 – bluetooth beacon)  Reducing the amount of false alarms. This was addressed by software update to AI analytics from object detection. New technology snagging. (P25 – AI Camera)  Setting the criteria for alarm (P59 – other: Video Analytics, AI object classification, Radar)  The bridge is a footpath / long distance footpath and telling the police everytime the sensor has been triggered is not helpful (P68 – other: LoRaWAN People Counter)  Tweaking the system to lessen false alarms (P106 -AI camera) | 8 | 5 | 1 |  |  |  | 1 | **15** |
|  |  |  |  | 1 | 6 | 3 |  | 5 | **15** |
| Installation difficulties | Power to remote locations (P9 – other: technology to digitally observe electronic devices)  Ability to quicky install due to health and safety (P9 –other: technology to digitally observe electronic devices)  Some locations are extremely expensive to make changes to due to engineering complexities (P13 – MA CCTV)  The technology is bulky and often requires a possession to install (P77 – AI Camera)  Coastal and remote countryside sites are often located far away from power infrastructure and have poor mobile phone reception. They are often much larger in scale and can be many miles long. Care should be taken to separate such sites from urban 'built structures' and not transfer conclusions. (P12 – additional comments) | 1 | 1 |  |  |  |  | 1 | **3** |
|  |  |  |  |  | 1 | 1 |  | 1 | **3** |
|  |  |  |  |  |  |  |  |  |  |
